# Bispecific T-Cell Engagers and Chimeric Antigen Receptor T-Cell Therapies in Glioblastoma: An Update

**DOI:** 10.3390/curroncol30090619

**Published:** 2023-09-15

**Authors:** Roa Alsajjan, Warren P. Mason

**Affiliations:** 1 Division of Medical Oncology and Hematology, Princess Margaret Cancer Centre, University Health Network, University of Toronto, Toronto, ON M5G 2C1, Canada; 2 Division of Neurology, Department of Medicine, College of Medicine, King Saud University, Riyadh 11461, Saudi Arabia

**Keywords:** glioblastoma, chimeric antigen receptor, CART, bispecific antibodies, bispecific T-cell engager, BiTE

## Abstract

Glioblastoma is the most common malignant primary brain tumor in adults. The prognosis is extremely poor even with standard treatment of maximal safe resection, radiotherapy, and chemotherapy. Recurrence is inevitable within months, and treatment options are very limited. Chimeric antigen receptor T-cell therapy (CART) and bispecific T-cell engagers (TCEs) are two emerging immunotherapies that can redirect T-cells for tumor-specific killing and have shown remarkable success in hematological malignancies and been under extensive study for application in glioblastoma. While there have been multiple clinical trials showing preliminary evidence of safety and efficacy for CART, bispecific TCEs are still in the early stages of clinical testing, with preclinical studies showing very promising results. However, there are multiple shared challenges that need to be addressed in the future, including the route of delivery, antigen escape, the immunosuppressive tumor microenvironment, and toxicity resulting from the limited choice of tumor-specific antigens. Efforts are underway to optimize the design of both these treatments and find the ideal combination therapy to overcome these challenges. In this review, we describe the work that has been performed as well as novel approaches in glioblastoma and in other solid tumors that may be applicable in the future.

## 1. Introduction

Glioblastoma (GBM) is the most common and the most aggressive form of primary brain neoplasm. The prognosis is very poor, even with treatment, which currently consists of maximal feasible surgical resection and radiotherapy with concurrent and adjuvant temozolomide [1]. This has been the standard treatment since 2005, prolonging survival by months, with most studies defining “long-term survival” in glioblastoma as longer than 24 months [2]. Recurrent disease usually portends a very poor outcome [3], with little survival benefit gained with the currently available second-line treatments such as bevacizumab and lomustine [4].

Breakthroughs in immunotherapy in cancer treatment have garnered great enthusiasm to trial these agents in glioblastoma. Although immune checkpoint inhibitors (ICI) have transformed treatment for some cancers, such as melanoma, results have been disappointing in GBM [5]. This can be explained by the fact that while ICIs aim to reverse the anergy or exhaustion of tumor-infiltrating cells (TILs), these are scarce in GBM and have lower expression of commonly targeted checkpoint receptors such as PD-1 and PD-L1. The location and biology of GBM pose unique challenges to the development of effective therapies. Firstly, the blood–brain barrier (BBB) forms a physical barrier to drug delivery. Secondly, the tumor microenvironment, rich in immunosuppressive cells such as myeloid-derived suppressor cells (MDSCs), tumor-associated macrophages (TAMs), regulatory T-cells (Tregs), and cancer-associated fibroblasts (CAFs), as well as anti-inflammatory cytokines such as tumor growth factor (TGF-β) and interleukin-10 (IL-10), forms a second physical barrier and renders the tumor immunologically “cold” and resistant to immunotherapy. Finally, the shifting antigenic landscape, both temporally and spatially, makes choosing an ideal target difficult and allows for tumor escape [6,7].

Chimeric antigen receptor (CAR) T-cell therapy and bispecific antibodies (BsAbs) are emerging therapies that rely on redirecting T-cells for highly specific and potent targeting of tumor cells as their mechanism of action. Here, we review the key points pertaining to the design and mechanism of action of each of these therapies, their advantages, the preclinical and clinical experience in GBM so far, some of the challenges of their design and use, and novel approaches to possibly overcome those.

## 2. Bispecific Antibodies

### 2.1. What Are Bispecific Antibodies

#### 2.1.1. Structure

**Figure 1:**
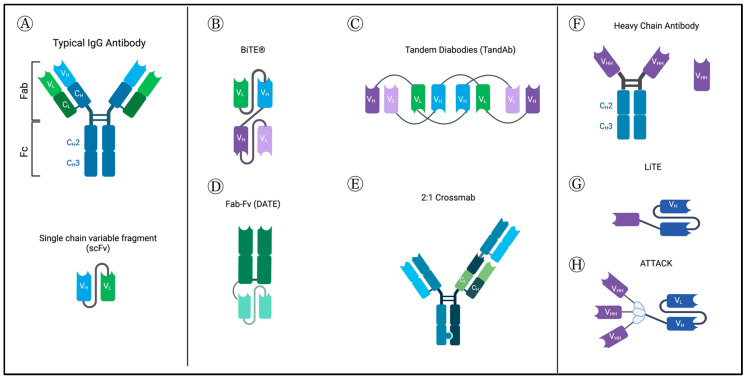
Normal antibody structure and different BsAb-platforms, as described in GBM studies. (**A**) Normal antibody structure (**top**) and structure of single-chain variable fragment (scFv). (**B**) Tandem single-chain variable fragment or Bispecific T-cell Engagers (BiTE^®^) consist of two single-chain variable fragments (scFv) connected by a flexible glycine–serine linker region. They most commonly target CD3ε subunit of the T-cell receptor (TCR) and a tumor-associated or tumor-specific antigen (TAA/TSA). (**C**) Tandem diabodies are tetravalent antibodies formed of 4 single-chain variable fragments (scFvs) linked together, providing two binding sites for each of two antigens, meaning it binds each target bivalently, similar to classic antibodies [9]. They have a molecular weight of about 105 kDa [12]. (**D**) Dual Antigen T-cell engagers (DATES) consist of a TAA-targeting Fab fragment connected to a CD3 targeting scFv. (**E**) Crossmabs are formed by exchanging one side of the CL with CH; by swapping the regions of one side heavy chain and light chain, the BsAb light chain can be assembled correctly. This platform allows trivalent and tetravalent BsAbs structures to be generated [10]. (**F**) Camelid heavy chain antibodies include an Fc portion and the variable heavy domains or single-domain antibodies (sdAb) or nanobodies. (**G**) Novel light T-cell engagers (LiTEs), which consist of single-domain antibodies linked to a CD3-scFv. (**H**) Novel ATTACK (Asymmetric Tandem Trimerbody for T-cell Activation and Cancer Killing) 3 + 1 design. Created with BioRender.com (accessed on 4 September 2023).

Normal antibodies are formed by two light chains, containing two domains, and two heavy chains, containing four domains. The two heavy chains are connected to each other (Figure 1A). The light and heavy chains are composed of variable and constant regions, and the antigen binding part is formed by a combination of the light and heavy variable regions and is part of the Fab fragment of the antibody. The Fc fragment interacts with effector molecules and cells. Normal antibodies bind one epitope bivalently [8].

Bispecific antibodies, as the name suggests, are antibody constructs with the ability to bind two antigens or epitopes. There are more than 23 different constructs being studied in trials, some that are IgG-like, meaning that they contain the Fc region, and some that do not. The removal of the Fc region significantly reduces the size of the molecule [9] but also eliminates Fc-dependent functions such as NK cell killing [10].

Tandem single-chain variable fragments or Bispecific T-cell Engagers (BiTE^®^) consist of two single-chain variable fragments (scFv) connected by a flexible glycine–serine linker region. They most commonly target the CD3ε subunit of the T-cell receptor (TCR) and a tumor-associated or tumor-specific antigen (TAA/TSA). This results in engaging the T-cell to the tumor cell, causing activation and subsequent tumor cell lysis [11]. Other platforms have also been used, as shown in Figure 1 below.

#### 2.1.2. Mechanism of Action

Their dual specificity allows for the use of BsAbs in many ways, such as the recruitment of immune cells or the blocking of immune checkpoint receptors, inflammatory factors, or dual signaling pathways. As mentioned above, the ability to engage immune cells is of specific interest. Normal cytotoxic T-cells (CTLs) are activated by a series of signals. Antigen fragments presented by MHC I molecules on tumor cells or MHCII on antigen-presenting cells (APCs) are recognized by the T-cell receptor (TCR) antigen binding site, which is covalently bound to a CD3 subunit, with CD8 acting as a co-receptor. This triggers intracellular signaling through the CD3ε subunit to activate the T-cell. A co-stimulatory signal from CD28 is necessary for cytotoxicity; otherwise, CD8+ T cells become anergic and undergo apoptosis [13]. Most immune cell-engaging bispecific antibodies act by binding the CD3ε subunit on T-cells and a TAA on the tumor cell to form a cytolytic synapse. This bypasses the need for MHC presentation, directly triggering activation signaling leading to the release of the pore-forming perforin and cytotoxic granzyme-B (GzmB) and, ultimately, apoptosis of the target cell [14].

### 2.2. Advantages

#### 2.2.1. BsAbs Are Easily Manufactured

Bispecific antibodies, especially BiTEs, are more easily produced by inducing mammalian cell lines to secrete the single chains, which allows for their production in large quantities. This is in contrast to CAR-T cell therapy, which requires the engineering of autologous T-cells and their expansion in the lab before they can be administered, a time and cost-intensive process. This could allow BsAbs to be more readily available and standardized in dosing and manufacturing [15,16,17,18].

#### 2.2.2. Smaller Size of BsAbs Fragments May Allow for Better Penetration of Tissue and a Higher Affinity Immune Synapse

For non-IgG-like BsAbs such as BiTE, the elimination of the Fc region significantly reduces the size of the molecule (<60 kDa vs. 130 kDa for the typical antibody) [19]. The elimination of the Fc portion also eliminates functions such as antibody-dependent cellular cytotoxicity (ADCC) and complement-dependent cytotoxicity (CDC) and the associated risk of cytokine release syndrome (CRS), which was observed with systemic administration of the IgG-like, EpCAM-targeting BsAb Catumaxomab [20,21].

Smaller molecular size may allow for better penetration into solid tumors [22], and it also allows for proximity between the T-cell and tumor cell with a higher affinity link, anchoring them together and increasing the likelihood of additive cytotoxicity, a process in which repetitive sublethal hits delivered by CTLs sequentially ultimately results in cytotoxic killing, and which usually would require a large number of encounters with different CTLs [23].

#### 2.2.3. BsAbs Are Highly Potent

Bispecific antibodies have superior potency to monoclonal antibodies (mAbs). For example, when compared to the mAb antibody rituximab, a CD19 × CD3 single-chain bispecific antibody was found to have a 100,000-fold higher in vitro efficacy and at very low concentrations of 50 pg/mL or less [14]. Similarly, an EGFRvIII-targeting bispecific antibody had a higher killing ability at a very low concentration of 0.01 microg/L compared to its parental EGFRvIII-mAb. This is possibly due to the fact that the monoclonal antibody cytotoxicity is mediated by its Fc portion, through ADCC and CDC, recruiting natural killer (NK) cells and macrophages, and once these mechanisms are depleted, no further cytotoxicity is observed [24].

The basis for this enhanced potency is thought to be the initiation of serial killing of tumor cells by the same T cell. The presence of BiTE alters T-cell behavior, causing T-cells to focus on a small area instead of scanning a large one and to have less but more prolonged direct contact with target cells sequentially, ultimately leading to their lysis. This was possible at low effector to target (E:T) cell ratio of 1:5 and at low concentrations [25].

#### 2.2.4. BiTE Mediated T-Cell Activation Is Independent of TCR-MHC Interaction

The lack of reliance on MHC-dependent antigen presentation has multiple implications in addressing immune escape. First, it avoids MHC downregulation causing antigen loss [26]. Second, TCRs are usually antigen-specific, but given BiTEs target the CD3e unit and cause direct activation of signaling pathways, they could activate T-cells with TCRs specific for antigens other than the target TAA [27]. Third, in addition to their proven ability to redirect both CD4+ and CD8+ T-cells, they have the theoretical ability to redirect any CD3+ T-cell. Despite this potential, activation of T-cells is highly dependent on dual binding, requiring the presence of both CD3 and the TSA/TAA. No activation of T-cells or cell lysis was observed when treating cells or tumors lacking the target antigen, indicating the specificity, and therefore, safety, of bispecific antibodies [24].

#### 2.2.5. BiTEs Initiate Bystander Tumor Cell Killing

Tumor antigen heterogeneity is a significant obstacle to designing therapies for solid tumors such as GBM. BiTEs can potentially overcome this as studies have found that while BiTEs only activate T-cells in the presence of TAA-positive tumor cells, cytokines released locally by BiTE-activated T-cells, such as TNF-α, IFN-γ, and FAS ligand seem to upregulate receptors such as intercellular adhesion molecule-1 (ICAM-1) and FAS on TAA-negative cells. ICAM-1 promotes the adherence of T-cells to tumor cells and has been found to provide an alternative costimulatory signal that restores the cytotoxic function of CTLs [28], and FAS activates the caspase apoptosis pathway. Together, these factors seem to render TAA-negative bystander cells more prone to cytotoxicity, leading to their apoptosis. Killing of TAA-negative cells depends on the dose of BiTE and the ratio of TAA-positive to TAA-negative cells. The degree of bystander tumor cell lysis also increases with the addition of TNF-α and IFN-γ [27].

#### 2.2.6. BiTEs May Overcome Immunosuppression by Redirecting Tregs

Tregs play a key role in disrupting anti-tumor immunity by suppressing immune effector cells and proinflammatory cytokines. However, treatments aiming to deplete them could result in the depletion of other T-cell populations and, when successful, usually have a transient effect. Therefore, therapies that could reprogram these cells may have better efficacy. Tregs counteract other immune cells by multiple mechanisms such as cytokine deprivation, IL-10 secretion, and TGF-B expression [29]. A study found that they could lyse immune cells via the perforin–granzyme B pathway and stipulated that this mechanism could be co-opted to kill tumor cells. Indeed, it found that Tregs redirected by an EGFRvIII-bispecific antibody seemed to show upregulation of granzymes and displayed effective cytotoxic lysis of tumor cells in vitro, and a later study of CAR T cells secreting BsAbs showed that when untransduced T cells were replaced with purified Tregs, cytotoxicity lysis of tumor cells still occurred [30,31]. Of note, this has been disputed by another study showing enhanced tumor growth upon administration of BiTE-activated Tregs in vivo [32,33]. Further validation in vivo is, therefore, required.

### 2.3. Targets

Choosing the appropriate target is one of the main challenges in solid tumor treatment in general and glioblastoma in particular. The ideal target would be a ubiquitously expressed surface antigen, which is exclusively expressed by the tumor and not present in healthy tissue. This would increase the chance of complete eradication while eliminating the risk of “on-target, off-tumor” toxicity. Glioblastoma is notorious for antigen heterogeneity, and no such target has been found to date. Nonetheless, there are multiple promising candidates under investigation. Table 1 provides an overview of the targets for both BsAbs and CART. Over the next few sections, we discuss the experience with them so far and some of the strategies aimed at overcoming antigen heterogeneity.

### 2.4. Preclinical Experience

#### 2.4.1. T-Cell-Engaging BsAbs

The most extensively studied and only one that has progressed to clinical trials is BsAbs against Epidermal Growth Factor Receptor variant III (EGFRvIII).

Choi et al. designed the first murine tandem scFv bispecific and were able to show in vivo efficacy using an orthotopic xenograft. Daily intravenous injection (IV) of bscEGFRvIII × CD3 achieved complete cure in six out of eight mice at low concentrations (1 microg/d), a dose approximately equivalent to 0.02 mg/kg in humans, low E:T ratios of 2.5:1, and without the need for co-stimulation. A significant antitumor effect was also achieved with very late-stage established tumors. The use of an EGFRvIII blocking soluble peptide completely inhibited bscEGFRvIII × CD3 activity in vitro and reduced it in vivo, providing further evidence for high specificity and a possible antidote should toxicity occur [40]. These experiments were carried out on immunocompromised mice, which is not ideal as it cannot fully simulate immune interactions and likely leads to an underestimation of the effect of treatment due to the lower number of T-cells and shorter half-life as compared to a functioning immune system [103]. Furthermore, this study showed the viability of systemic therapy for intracranial tumors. This may be explained by exclusive expression of EGFRvIII intracranially, as opposed to targeting of less specific antigens, which may result in peripheral accumulation of the antibodies [104,105].

Gedeon et al. developed the first fully humanized antibody fragments for increased safety, with a focus on choosing fragments without cross-reactivity with wild-type EGFR. Again, systemic administration of hEGFRvIII-CD3 bs-scFv showed a significant survival benefit in multiple in vivo models, even for more established tumors, effectively curing them. The survival was reported to be about 80% [41]. These striking results, despite tumor antigen heterogeneity, could be due to the essential role of EGFRvIII in maintaining EGFRvIII negative cells and its presence on glioblastoma stem cells (GSCs) which give rise to those, or due to the bystander effect demonstrated in other studies [27]. The group later performed a first-in-human dose calculation using a minimal anticipated biological effect level (MABEL) approach detailing the method for calculating a maximum safe recommended starting dose of 57.9 ng/kg specifically for humanized EGFRvIII × CD3 bi-scFv [106].

Ellwanger et al. developed a TandAb with two binding sites for EGFRvIII in the core and two binding sites for CD3 in the external positions. This construct has two advantages: it binds its targets bivalently and has a molecular weight of >100 kDa, exceeding the first-pass renal clearance threshold and resulting in a longer half-life. EGFRvIII-binding affinities were found to be >10-fold that of monovalently binding scFv. In vitro, TandAb showed superior cytotoxicity compared to previously studied BiTEs. This seemed to correlate to higher CD3 affinity. While higher CD3 affinity has been shown to indeed improve cytotoxic function, especially in conditions with reduced density of tumor-specific antigen (TSA), one concern with CD3 bivalent—and therefore higher affinity—binding is an increase in cytokine release. Furthermore, multiple in vivo studies comparing different CD3 affinities showed no significant effect on potency. On the contrary, it seems that factors other than affinity, such as pharmacokinetics and rate of internalization into T-cells, may even favor lower affinity [107,108,109]. In vivo, treatment with TandAb showed a significant reduction in tumor volume in a dose-dependent manner compared to control, but not reaching statistical significance when compared to the mAb Cetuximab. This study was limited by the use of a subcutaneous xenograft model and the need to terminate the experiment on day 27 due to disease complications [12]. Pharmacokinetics and half-life were not reported in detail. So, while in vitro data show superiority to previous BiTE constructs, the significance of these findings is unclear.

To address the challenge of the short half-life of these molecules, two studies were performed using larger, IgG-like constructs. Sun et al. designed an EGFRvIII-targeting IgG-like bispecific antibody with attenuated Fc-related functions to prevent ADCC and CDC and decrease the risk of infusion reactions while preserving IgG pharmacodynamics. Their final antibody had a molecular weight of 145 kDa. This bispecific antibody was highly effective in an immunocompromised in vivo model, but this was a subcutaneous model, and the effect of the higher molecular weight on intracranial delivery was not verified. This study again showed that a slightly higher E:T ratio of 2:1 and a higher dose were more effective at producing a complete response without recurrence, although a dose as low as 0.33 mg/kg (five times lower compared to the mAb) also showed the ability to reduce tumor size. The main goal of prolonging half-life was achieved as it had a surprisingly longer half-life of ~14 days compared to ~3.5 days for the monoclonal antibody [24]. Iurlaro et al. utilized the Crossmab platform to produce a 2:1 format with two EGFRvIII-binding Fabs and one CD3-binding Fab with a modified Fc. In vivo testing of intravenous administration in an orthotopic patient-derived xenograft (PDX) model yielded impressive results with eradication of tumors in some cases, providing evidence for the efficacy of systemic administration of these larger constructs for CNS tumors. These promising results supported a phase 1 trial for this EGFRvIII T-cell-bispecific antibody (TCB) (NCT05187624) [42].

Interleukin-13 receptor subunit alpha-2 (IL13Rα2) is another attractive target as it is expressed at a higher frequency of 40–76% in IDH-wildtype GBM, but systemic delivery carries a higher risk of complications due to its minimal expression on normal tissue [59,60,61,62,63,64]. Bispecific antibodies targeting IL13Ra2 were tested as a DNA-launched bispecific T-cell engager (BTE) and as BsAbs secreted by neural stem cells, described in the next sections.

Fibroblast growth factor inducible 14 (Fn14) is another highly expressed, relatively specific target. In a preclinical study [73], Fn14 × CD3 BiTEs showed similar results to those described in EGFRvIII BiTE studies, successfully suppressing tumor growth after intracranial injection. Local delivery was chosen because of the short half-life of the tandem scFv design and the presence of Fn14 on normal tissue.

The dual antigen T-cell engager (DATE) platform (Figure 1D) was used in two studies to target carbonic anhydrase (CA9) [77] and CD133 [74], which are thought to mark a stem cell-like population of tumor cells. CA9 is expressed in normal gastrointestinal cells, and CD133 is expressed on a variety of stem cells, including hematopoietic. Although DATEs targeting CA9 or CD133 showed a potent cytotoxic effect in vitro, they failed to achieve a sustained survival benefit in GBM mouse models. The authors proposed that this was due to short half-life and suboptimal dosing.

#### 2.4.2. BsAbs with Targets Other Than CD3e

Targeting NKGD2 ligands is problematic because tumor cells can downregulate those or shed them proteolytically, with the shed material blocking effector cells from recognizing tumor cells. Lymphocytes and natural killer cells (NKs) can be engineered to express NKG2D in a CAR fashion, linking the extracellular domain of NKG2D to a signaling molecule such as CD3ζ, and BsAbs could then activate them through a TAA/TSA rather than NKG2D-L. A study combining a tetravalent bispecific antibody targeting HER2 (Erb2) and NKGD2 with NKG2D-based CARs (NKAR) showed impressive suppression of tumor growth in a murine model of GBM with low expression of NKG2D-L [79].

#### 2.4.3. BsAbs Targeting Dual Signaling Pathways

Aside from T-cell redirection, other mechanisms of action of BsAbs, such as blocking redundant signaling pathways, have been explored in GBM. A study utilizing a bispecific variable heavy domain antibody to target both EphA2 and EphA3, members of the Eph receptor family, which are found in developing but not adult tissues and are enriched in glioblastoma and especially GBM stem cells (GCS), showed decreased tumorigenesis and increased differentiation in a recurrent BM model. Targeting only one of these receptors was not sufficient to produce a response [84].

### 2.5. Clinical Experience

#### 2.5.1. Clinical Experience in Other Malignancies

Blinatumomab is a first-in-class CD19-targeting BiTE and the first to be approved for cancer therapy after showing remarkable response rates of almost 70% in CD19-positive, relapsed/refractory (R/R) hematological malignancies. Its main challenges were the short half-life requiring continuous IV infusion to achieve stable plasma levels and, therefore, clinical effect, and toxicity at higher doses. Long-term follow-up studies showed an impressive median OS of 5.8 years for lymphoma, but treatment at the maximum effective dose is necessary to achieve this substantial survival advantage [16,110].

Tebentefusp is a bispecific protein targeting gp100 which recently obtained FDA approval in January 2022 after a phase 3 trial showed a survival benefit for HLA-A*02:01–positive patients with metastatic uveal melanoma, increasing 1-year survival from 59% to 73% [111]. There are over 45 registered clinical trials of T-cell engagers targeting a variety of other solid tumor-associated antigens. While most have preliminary evidence of efficacy, important limitations are the narrow therapeutic index due to on-target off-tumor toxic effects and the short half-life requiring continuous infusion for days [112,113,114,115,116,117,118,119,120].

#### 2.5.2. Glioblastoma

Etevritamab (AMG 596) is an EGFRvIII targeting BiTE studied in a first-in-human, open-label, sequential dose-escalation and dose-expansion trial (NCT03296696) for recurrent or newly diagnosed GBM or malignant glioma after a preclinical study showed successful treatment of intracranial tumors and included a toxicology study showing no evidence of toxicity in cynomolgus monkeys even at doses of 450 microg/kg/d [121]. The first clinical data for 14 recurrent GBM patients treated through continuous IV infusion reported no dose-limiting toxicities (DLT), but serious adverse events (SAE) occurred in 50%, and the most common were headache and altered level of consciousness. Out of eight evaluable patients, one had a partial response (PR), and two had stable disease (SD). Preliminary pharmacokinetic data confirmed an increase in steady-state serum exposures proportional to dose [122,123].

BRiTE is a humanized EGFRvIII targeting bi-scFv which will be trialed in patients with grade 4 malignant glioma (NCT04903795). This will be administered as a single bolus, either as monotherapy or with peripheral T-cell infusion, after completion of SOC chemoradiation and at least six cycles of temozolomide or at recurrence, followed by 28 days of monitoring for CRS [15].

RO7428731 is a T-cell-bispecific antibody (TCB) targeting EGFRvIII utilizing the 2:1 Crossmab platform described in the preclinical study by Iurlaro et al. [42]. This is also being studied in an open-label, non-randomized safety and tolerability trial for patients with newly diagnosed GBM. The treatment is administered as maintenance after completion of standard-of-care. The trial started enrolling in April 2022 and is estimated to be completed in February 2025.

### 2.6. Challenges and Novel Approaches

#### 2.6.1. Delivery

The method of delivery of bispecific antibodies influences pharmacokinetics, the ability to achieve full penetration into tumor tissue, and the risk of systemic toxicity. Classic bi-scFv (BiTE) design has a terminal half-life of about 2.5 h [124] and necessitates continuous infusions. Therefore, aside from altering the pharmacokinetics via designing larger molecules or the addition of a modified Fc portion, immune and other types of cells or oncolytic viruses could be engineered to locally secrete BsAbs within the tumor.

**DNA Launched Bispecific T-cell Engagers (dBTE)**. By designing a synthetic DNA plasmid expressing the desired BsAbs and injecting them intramuscularly, followed by electroporation at the site, Bhojnagarwala et al. were able to significantly increase the persistence of an IL13Ra2 × CD3 BTE. Peak cytotoxicity after administration of the dBTE was observed on day 7, lasting until day 13, with T-cell activation and cytokine expression detected until day 19. In contrast, recombinant BTEs administered systemically were cleared from the serum by day 5. This seemed to also correlate with enhanced cytotoxicity, possibly due to overall increased levels of BTE at the tumor site. This method of delivery also proved effective in an intracranial model, and dBTE resulted in the elimination of 5/9 tumors after a single injection [65]. This effect was also observed in HER2-targeted DNA-launched BTE, persisting 4 months after a single injection [125].

**Neural stem cells**. Neural stem cells (NSCs) have the advantage of being native to the brain, able to migrate to intracranial tumors in animal models, and to survive in a hypoxic environment such as that produced by GBM. Pituch et al. intracranially delivered modified NSCs secreting BiTEs targeting IL13Ra2, which were detected up to 7 days post-administration, prolonging survival by 63%. However, NSCs decreased over time, with only a few NSCs near the third ventricle detected at 90 days, suggesting that repeated administration would still be needed [66].

**Bispecific Antibody Armed Activated T-cells (BATs)**. Arming-activated T-cells with a bispecific antibody targeting a TAA and CD3 could theoretically overcome some of the limitations of CAR T-cell therapy, such as the time-intensive manufacturing process while bypassing the need for BsAbs to interact with other T-cell populations. An in vitro study of a tetravalent recombinant EGFR bispecific (rEGFRbi) antibody targeting wild-type (wt) EGFR showed similar efficacy to other platforms [34], and a previous small phase 1 trial in pancreatic cancer showed durable responses in patients with no dose-limiting toxicities [126]. There is currently an ongoing phase 1 trial (NCT03344250) of EGFR BATs for newly diagnosed GBM, administered after SOC chemoradiation weekly with temozolomide. The targeting of EGFR rather than EGFRvIII may also address antigen heterogeneity and immune escape.

**BiTE-secreting genetically engineered macrophages (GEMs)**. Macrophages have a propensity to accumulate in tumor tissue and have the added advantages of antigen presentation to T-cells, secretion of cytokines that support T-cell function, and ability to clear debris [127]. Because they do not proliferate, this may limit possible toxicity and unchecked protein secretion. In a preclinical study, macrophages secreting EGFRvIII-targeting BiTEs induced a more robust activation of T-cells compared to BiTE alone. Intratumorally injected BiTE-secreting GEMs delayed tumor growth early on, but tumors later rebounded with no significant extension of survival. However, when the GEMs were transduced with BiTE and IL-12, tumor growth was prevented for 36 days, whereas BiTE alone or combined with IL-12 delayed but did not prevent growth, suggesting an added benefit of GEMs [45].

**Oncolytic viruses expressing bispecific antibodies**. Oncolytic viruses (OVs) are a very attractive option for multiple reasons. Infection of cells relies on tumor-specific changes, and effects are, thus, restricted to the tumor site. They then promote inflammation and an immunogenic response. Lysis of tumor cells may further lead to antigen shedding, antigen presentation to T-cells, a systemic antitumor effect, and, ultimately, epitope spreading. OVs can be engineered to secrete other immunomodulators, such as cytokines or ICIs. OVs produce an immunogenic response, recruiting T-cells to the tumor, which could then be optimally redirected by the BiTE [128]. In a preclinical study of pediatric high-grade gliomas, Arnone et al. interestingly employed two viral therapy platforms: oncolytic adenovirus (OA) and gene therapy delivering EphA2 targeting BiTE by a second virus (EAd). The authors postulated that this might result in broader targeting for a heterogeneous tumor as cells resistant to infection by the OA may be infected by the EAd while decreasing the risk for off-tumor toxicity as simultaneous infection is less likely in normal tissue. Intra-tumoral amplification of the BiTE could bypass the limitations of systemic delivery and short half-life. Indeed, they were able to show persistent T-cell activation at 60 days, correlating with improved responses in the combined treatment group [129].

#### 2.6.2. Antigen Heterogeneity and Antigen Escape

Antigen loss has been a main challenge in T-cell engaging therapy, occurring in about 30% of patient in studies of blinatumomab and accounting for treatment failure [130]. Strategies to overcome this include targeting a more widely expressed antigen or multiple antigens at once.

While EGFRvIII is very attractive as a target because it is very specific to malignant tissue, as mentioned, there is significant variability in its expression, and it is only present in about 30% of glioblastomas. Wild-type EGFR, on the other hand, is amplified in about 80% of glioblastomas, but targeting it carries a very high risk of on-target, off-tumor toxicity to skin, lungs, and gut. To address this specific challenge, Choi et al. [31] designed EGFRvIII-targeting CAR T-cells, which secrete EGFR-targeting BiTEs locally at the tumor site once activated, as EGFR is not expressed by normal brain tissue. CART.BiTE, thus, has the ability to target multiple antigens, one of which is very prevalent in glioblastoma, and recruit bystander effector cells. In a preclinical model, the treatment was delivered intraventricularly and resulted in complete and durable responses in all mice. CAR T-cell migration outside the CNS tended to occur when the tumor did not express the target antigens. BiTEs had the capability of recruiting transduced and un-transduced cells. The combination of BiTE and CART produced T-cell proliferation over a longer period compared to stimulation by BiTE alone (30 d vs. 12 d). This is hypothesized to be due to distinct T-cell differentiation, whereas BiTE promotes effector memory cells (T_EM_), CART or CART.BiTE promote less differentiated central memory cells (T_CM_). The combination treatment was also superior in terms of exhaustion markers, whereas PD-1, TIM-3, and LAG-3 were associated with BiTE alone and treatment with CART.BiTE seemed to have the opposite effect. In vivo, CAR-EGFRvIII.BiTE-EGFR was found to produce a response even in EGFRvIII-negative tumors. A skin graft toxicity model was used to assess the safety of targeting wild-type EGFR. Treatment was administered intravenously to increase sensitivity, and while mice treated with CART-EGFR showed T-cell infiltration of grafts and evidence of cutaneous graft vs. host disease, those signs were absent in mice treated with CART.BiTE, where BiTE was secreted at low concentrations. BiTE was also not detected in the peripheral blood [31].

Another possible approach is the administration of BTEs targeting different antigens. In a study of DNA-launched BTEs against EGFRvIII and HER2, which are expressed in 30% and 60% of GBMs, respectively, the combination of EGFRvIII-DBTE and HER2-DBTE resulted in 80% survival in a heterogenous GBM mouse model, vs. 20% and 10% for the EGFRvIII and HER2 treatment groups [44].

#### 2.6.3. Immunosuppressive Microenvironment and T-Cell Exhaustion

There is a myriad of possible mechanisms to modulate the TME as many different cytokines, chemokines, cells, and receptors are involved in maintaining immune suppression [6]. Because of its complexity, monotherapy is unlikely to be adequate in reversing this immune suppression and, therefore, impacting survival meaningfully. There are several promising approaches that combine immunotherapies as a strategy for treating GBM.

The paucity of TILs is a significant barrier to the development of immunotherapy for GBM. The chemokine C-C motif ligand 5 (CCL5) is an inflammatory chemokine that promotes chemotaxis of immune cells to the tumor once an immune response is activated [131]. Tian et al. [132] designed a bispecific antibody consisting of a single-chain variable fragment of the EGFR mAb, Cetuximab, and CCL5 together on a functional Fc region and engineered an oncolytic herpes simplex virus type 1 (oHSV) to express it (OV-Cmab-hCCL5). They found that a single intracranial injection of this combination significantly prolonged survival compared to OV alone and even more so with two injections. Even without the presence of T-cells, OV-Cmab-hCCL5 was still significantly better at preventing progression, owing to the Fc-mediated actions of NK and macrophages.

Immune checkpoint blockade, while unsuccessful as monotherapy in glioblastoma, remains a logical solution to the problem of T-cell exhaustion, and targeting immune checkpoints is still under extensive study. Novel designs aiming to combine the bispecific engager design with immune checkpoint blockade include checkpoint inhibitory T-cell engagers (CiTE), such as the one developed by Hermann et al., by fusing the extracellular domain of PD-1 to a CD33 × CD3 BiTE. This resulted in the eradication of leukemia cells without observable immune-related adverse events (ir-AE) [133] and, therefore, may be a safer approach than administering BiTE in addition to monoclonal antibodies targeting ICI.

Another way of addressing T-cell exhaustion is the addition of a costimulatory signal, which has been well described in CAR T-cell therapy (see Section 3). In their study of OA + EAd, Arnone et al. demonstrated that the addition of CD28 resulted in more potent cytotoxicity, especially of more resistant cell lines, while maintaining T_CM_ and T_EM_ cells rather than terminally differentiated T-cells—contributing to their persistence [129]. Implementing this for clinical use is problematic, however, as previous studies with the systemically delivered CD28 super-agonist resulted in severe and unacceptable toxicity [134]. Simultaneous multiple interaction T-cell engagers (SMiTEs) consist of two BiTEs, with one targeting a TAA and CD3 and the other targeting CD28 and either the same antigen or a different target, such as PD-L1, turning an immune checkpoint receptor into one providing a costimulatory signal instead [135]. Another design combining targeting of CD3 and CD28 is the trispecific T-cell engager (TriTE), which effectively suppressed tumor growth in a myeloma mouse model and showed memory/effector T-cell proliferation and reduced regulatory T cells [136].

Oncolytic viruses also provide flexibility in designing combination therapies. Porter et al. attempted this by combining an oncolytic virus secreting a BiTE, IL-12, and PD-L1 inhibitor with CAR T-cells targeting a different antigen (CAd-Trio). This approach is attractive as it enhances delivery by an OV, targets multiple antigens via BiTE and CAR T cells, and modulates the tumor environment. Indeed, there was early improved control with the combination treatment compared to monotherapies. However, in the long term, there was loss of CAR expression and increased PD-L1, indicating exhaustion, with a loss of the initially observed survival benefit. This was postulated to be due to the inadequacies of animal models lacking a fully functioning immune system, the augmentation of which is the target of these therapies [137].

Another strategy for rendering the TME less hostile is by targeting immunosuppressive cells. Cancer-associated fibroblasts have been found to play a key role in tumor growth and metastasis, promoting angiogenesis and secreting TGF-B, among other immunosuppressive functions [138]. Targeting these cells is feasible through fibroblast-associated protein (FAP), but that could be problematic due to its presence in healthy fibroblasts. Therefore, it needs to be directly delivered to tumor tissue to avoid toxicity. This was achieved by an oncolytic virus encoding a FAP-targeting BiTE. Multiple studies investigating different viral platforms showed effective tumor lysis, depletion of CAFs and immunosuppressive cytokines, repolarization of macrophages to an M1 phenotype, and increased T-cell infiltration and activation correlating with improved survival in mouse models [128]. An ongoing trial (NCT04053283) is assessing a tumor-selective vector expressing a FAP-T cell activator bispecific antibody, which also expresses CXCL9, CXCL10, and IFN-a in multiple epithelial cancers, and preliminary data showed no toxicity [139]. Another phase 1 trial is testing this in combination with nivolumab (NCT05043714).

Similarly, tumor-associated macrophages could be targeted by OV-BiTE. Scott et al. engineered BiTEs and TriTEs targeting CD206 and folate receptor B (FR-B), which are upregulated on macrophages. A specific TriTE design, which includes an extra CD3 scFv, therefore bivalently binding CD3, and either of the target antigens showed targeted depletion of TAMs, preferentially M2 phenotype [140].

#### 2.6.4. Toxicity

Adverse effects are either related to T-cell activation and cytokine secretion, culminating in cytokine release syndrome (CRS) with or without neurotoxicity, or to off-tumor toxicity when the target antigen is expressed in normal tissues.

We have already discussed some methods used to enhance safety. Spatial control, such as with CAR.BiTE or OV-BiTE, enables the use of BiTEs targeting less specific antigens by ensuring their secretion only in the presence of other tumor markers. Using a 2 + 1 design alters the affinity of the BsAbs, requiring a higher density of the target antigen, which is usually found only on tumor cells, thereby increasing their ability to discern between tumor and non-tumor cells. Another way to alter the affinity is the arrangement of heavy and light chains. Forward arrangement (VL-VH-VH-VL) results in higher specificity for the target antigen, lower binding affinity to CD3, and lower levels of T-cell activation and cytokine release in the presence of TAA. Arranging the BiTE in the reverse order (VH-VL-VL-VH) by contrast resulted in T-cell activation in the presence of TAA-negative cells and off-target toxicity [65].

Building on the concept of spatial control, conditional BsAbs are engineered to only become active once conditions associated with the tumor are met, for example, by including a peptide mask that is cleaved by tumor proteases or by requiring the presence of two antigens for unmasking and dimerization of the CD3 binding domain [141,142,143,144,145,146,147]. These platforms are yet to be tested in GBM but would likely be applicable, especially if designed for activation in a hypoxic TME, for example.

#### 2.6.5. Novel Designs Utilizing Nanobodies

Heavy chain antibodies are present in the Camelidae family of mammals and consist of an Fc portion similar to a conventional antibody but with the two antigen binding sites consisting of a single-variable heavy chain (VHH) only [148]. Single-domain antibodies (sdAb) or nanobodies are formed of this region.

Nanobodies can offer several advantages over conventional antibodies or scFv.For one, they are easily produced as they can be expressed in bacteria.

But the most apparent advantage is their smaller size (15 kDa), which may afford better tissue penetration, especially through the BBB. A study comparing the utility of a fluorescent EGFR-targeting nanobody vs. cetuximab for optical imaging of tumors found significantly higher uptake of the nanobody in mouse models [149]. This smaller size can also lead to better access to difficult-to-reach antigens due to the presence of a flexible loop [150]. A logical drawback to the smaller size of nanobodies is increased renal clearance and, therefore, significantly shortened half-life. This could be overcome by half-life extended designs, including an anti-albumin nanobody [151] or local secretion by a variety of cell types, as discussed above.

SdAbs are also more stable compared to conventional antibodies because of the lack of the hydrophobic interactions between the VH and VL chains, which can lead to mispairing. This is especially relevant when designing bispecific antibodies, as two nanobodies targeting different antigens can be linked without much need for elaborate genetic engineering. Bivalent nanobodies can similarly be easily produced to increase the affinity without significantly increasing the size of the antibody (30–35 kDa) [19,148]. Biparatopic nanobodies target two different epitopes of the same antigen and have been shown to have superior efficacy [151].

As most scFvs are based on murine or chimeric mAbs, there is a risk of the formation of neutralizing antibodies. The effect of this on clinical efficacy is not immediately apparent, but the resulting immune response has been reported to decrease CAR T-cell persistence [152]. The same phenomenon has not been observed with the use of nanobodies [153].

Preclinical studies have shown the feasibility of nanobody use. Xing et al. constructed an IgG-like anti-HER2 bispecific antibody consistent of an anti-HER2 nanobody and anti-CD3 scFv, which is easier to produce and showed efficacy in vivo and in vitro [154]. Xie et al. described the novel light T-cell engagers (LiTEs), which consist of a single-domain antibody linked to a CD3-scFv. These have a molecular weight of slightly over 40 kDa. While these are small, their expression locally may be an advantage as their secretion outside of the tumor leads to rapid clearance and avoidance of toxicity [155]. The ATTACK (Asymmetric Tandem Trimerbody for T-cell Activation and Cancer Killing) design by the same group further expands on LiTEs by increasing TAA affinity through a 3 + 1 design. This resulted in a dramatic increase in efficacy and potency, requiring lower concentrations compared to LiTE. This low-affinity multivalent antibody design could enable discrimination between cancerous and non-cancerous cells, therefore increasing safety [156].

## 3. CAR T-Cell Therapy

### 3.1. What Are CAR T-Cells

#### 3.1.1. Design

**Figure 2:**
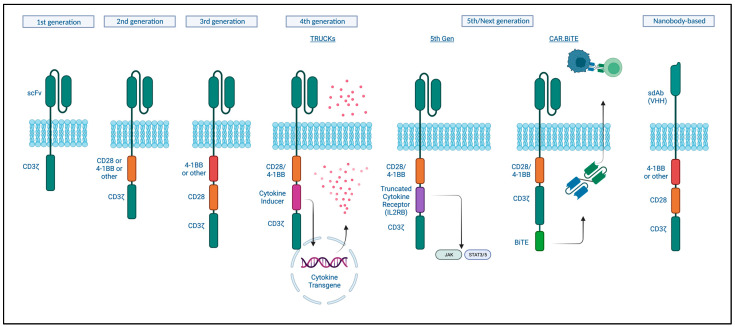
Basic CAR structure and different CAR T-cell generations. Created with BioRender.com (accessed on 4 September 2023).

Chimeric antigen receptor (CAR) T-cells are T-cells that are genetically engineered to express a receptor like structure that recognizes a tumor-specific or tumor-associated antigen in an MHC1-independent manner in order to redirect T-cell cytotoxic activity to those tumor cells. The CAR consists of an antibody-like surface domain, a transmembrane domain, and an intracellular signaling domain (Figure 2) [157]. The antibody-like domain is formed by a single-chain variable fragment (scFv) recognizing a specific antigen. The intracellular signaling domain has been the focus in enhancing CAR T function.

First-generation CARs engineered in 1993 only combined a portion of an antibody with the CD3ζ subunit for intracellular signaling. However, these did not prime resting T- cells, and the cells did not persist and so were not clinically effective [158]. Second-generation CARs contained a costimulatory signaling domain, either CD28 or 4-1BB, and this enhanced T-cell activation, cytokine release, and improved persistence of these cells in the circulation [159,160]. Current approved CAT-cell therapies are based on this design [161]. While these were shown to be effective at eradicating tumors, relapses were reported to be associated with a lack of persistence [162]. Third-generation CARs incorporate both of these costimulatory signals based on the hypothesis that these could act in a complementary manner. Indeed, improved expansion and longer persistence were observed; however, this did not seem to significantly improve on the second-generation designs when it came to clinical responses [161]. Fourth and next-generation CARs, so-called armored CAR T-cells, incorporate protein expression into a second or third-generation design to enhance T-cell function, modulate the immune environment, or improve toxicity. T-cells redirected for universal cytokine-mediated killing (TRUCKs) are engineered to express a transgenic cytokine to improve their function as well as ameliorate immune suppression. Multiple cytokines have been tested and are being tested in clinical trials, including IL-12, IL-18, and IL-7, among others, with or without chemokines [163]. Fifth-generation CAR-T cells include an intracellular cytokine receptor fragment; e.g., IL-2RB, capable of activating the JAK-STAT pathway, thereby prompting proliferation, decreasing terminal differentiation, and increasing cytolytic activity [164]. CAR T-cells could similarly be engineered to produce antibodies or antibody-like proteins, such as PD-1 scFv or scFv-Fc, for immune checkpoint inhibition or even bispecific antibodies (discussed in the previous section) [165]. To increase safety, a surface antigen could be incorporated, which then can be targeted by another pharmaceutical in case of toxicity to activate transgenes with the ability to “switch off” the CAR T-cell using different mechanisms [166].

#### 3.1.2. Manufacturing and Mechanism

**Figure 3:**
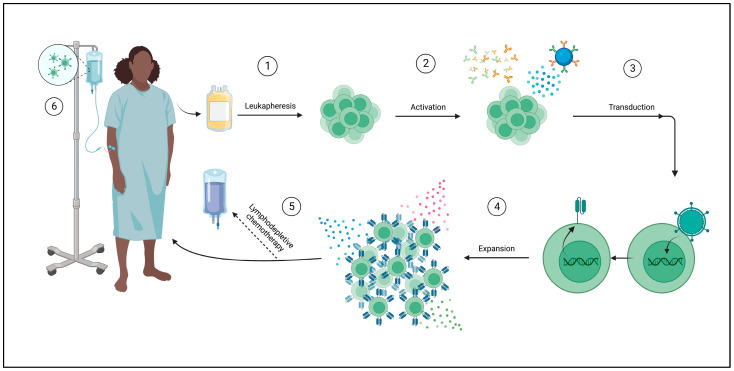
Autologous CAR T-cell manufacturing process. ① A peripheral blood sample is taken from the patient for leukapheresis, wherein T-cells are isolated. ② T-cells are then activated in a variety of ways: using anti-CD3/anti-CD28 antibodies or magnetic beads or through cellular activation using dendritic cells or artificial antigen-presenting cells (AAPCs). ③ T-cells are transduced with the desired CAR using a viral vector such as lentivirus, plasmid DNA trans-infection (not shown), or cytoplasmic mRNA introduction (not shown). ④ CAR T cells are expanded ex vivo to achieve therapeutic doses through a variety of methods, such as cytokines (shown) or AAPCs (not shown). ⑤ Lymphodepleting chemotherapy may be administered prior to infusion of the final product. ⑥ CAR T cells are delivered to the patient. Created with Biorender.com (accessed on 4 September 2023).

CAR T-cells are derived from the patient’s own lymphocytes. This process begins with collecting peripheral blood and then separating lymphocytes using leukapheresis. Further enrichment for a specific subtype, such as CD4+, CD8+, CD25+, or CD69L cells, can be performed. The next step is the activation of T-cells, which can be achieved by using anti-CD3 antibodies, CD3/CD28-antibody coated beads, or through dendritic cells or artificial antigen-presenting cells (AAPCs). T-cells are then genetically modified through a variety of methods. Gene transfer can be achieved through viral vectors, plasmid DNA transfection via transposon/transposase system, or in vitro transcribed mRNA introduction into the cytoplasm by electroporation or endocytosis. The most frequently used viral vectors are y-retrovirus and lentivirus. Expansion of the modified cells is then necessary to obtain therapeutic doses (Figure 3). This can be achieved by using bioreactors, multiple of which exist, or AAPC stimulation [157,167].

### 3.2. Advantages

**CAR T cells expand and effectively traffic to the tumor**, as evidenced by the presence of CAR T cells in the tumor after systemic administration of EGFRvIII-CART [57]. Their ability to traffic to distant sites after local administration was also proven on PET imaging [168]. The advantage here lies in the fact that an antitumor effect can be achieved without relying on endogenous immune cells, which are scarce and suppressed in tumors such as glioblastoma.

**CAR T cells mediate potent tumor cell killing independently of MHC antigen presentation**. In a study performed in CLL patients, it was estimated that one CAR T cell had the ability to kill 1000 tumor cells [169].

**CAR T cells have the potential for long-term engraftment**. A possible advantage of CAR T cells is the ability to engineer them to persist longer and theoretically prevent disease recurrence by providing ongoing surveillance. Enhanced persistence was achieved with second-generation designs due to the addition of a costimulatory domain, as mentioned. CD19-4-1BB-CD3ζ CAR T cells have been shown to persist for many months and even years in the circulation [170]. CAR T cells have been shown to display a central memory cell phenotype with high CCR7 expression after many months in the circulation [169]. Effector memory T cells are able to eradicate tumor cells but do not persist for long and will eventually terminally differentiate. Central memory cells, on the other hand, have a better replicative capacity and are able to mount a response upon encountering the target antigen again [171]. There have been ongoing efforts to identify the necessary manufacturing conditions for expanding a subset of memory cells with a less differentiated phenotype, as this has the potential to increase their efficacy while decreasing toxicity [172]. Stem-like memory T cells (TSCM-like) can self-renew and differentiate into TEM, TCM, or TEFF cells, and there has been significant interest in developing those for therapy. This has been achieved by inducing Wnt signaling or by use of IL-7 and IL-15 [171].

### 3.3. Preclinical and Clinical Experience

There are seven CAR T-cell therapies (CART) approved for hematological B-cell malignancies. This was based on remarkable responses in refractory disease with CD19-CART. The experience in solid tumors has not had the same trajectory thus far, which is not surprising when considering all the aforementioned additional challenges [173]. CAR T-cell therapy has reached advanced stages of testing for glioblastoma, with multiple phase 1 studies completed and many more underway (Table 2).

#### 3.3.1. Preclinical Experience

The earliest preclinical studies were conducted with CAR T cells targeting EGFRvIII, IL13Ra2, and HER2 and had impressive rates of tumor control and improved survival in murine models [47,49,50,67,80], leading to the design of multiple clinical trials. Other antigens tested in glioblastoma models, most of which have already made it into clinical testing as below, include EphA2, B7-H3, CD133, CD70, GD2, NKG2D, Fn14, and podoplanin (Table 1).

#### 3.3.2. Clinical Experience in Glioblastoma

Brown et al., focused on IL13Ra2 CAR T cells. They performed a first- in- human trial of CAR T-cells in three patients, delivering repeated treatments into the tumor cavity. This trial showed the feasibility of local delivery as well as the relative safety of CAR T cells with three grade 3 neurological events at the highest dose of 1 × 10^8^, which were transient [18]. The same group is conducting a phase 1 trial (NCT02208362) using an enhanced CAR T design to include a costimulatory domain, modified Fc-hinge to avoid off-target effects causing decreased persistence, and chose enriched central memory cells based on remarkable tumor regression after intracavitary followed by intraventricular infusion of this product in one patient [69]. Multiple other trials are ongoing to test intraventricular delivery for leptomeningeal disease, systemic delivery, and combination therapy with immune checkpoint inhibitors (see Table 2).

O’Rourke et al. reported on a phase 1 trial with CAR T cells targeting EGFRvIII. Eighty percent of the patients had received two or more lines of treatment and had multifocal disease. Despite systemic delivery, no EGFR toxicity, cytokine release syndrome (CRS), or typical immune effector-cell-associated neurotoxicity syndrome (ICANS) were observed. However, siltuximab and IL-6 antagonists and corticosteroids were administered in 2/10 patients for new neurological symptoms in the 1st month. Tocilizumab was not used as its BBB penetrance is questionable, and its mechanism of action of receptor blockage may increase overall CNS exposure to IL-6. Although IL-6 was high in these patients, it did not correlate with a high c-reactive protein (CRP) or clinical symptoms of CRS, such as hypoxia, hypotension, or fever. Neurosurgical intervention was necessary in 7/10 patients post-infusion, in 4 of whom this was determined prior to proceeding with infusion. Early operation post-infusion enabled examination of CAR T-cell trafficking to the tumor, while late surgery enabled clarification of persistence. CAR T-cells were detectable peripherally in all patients despite a lack of prior lymphodepletion, although some were lymphopenic at baseline. Levels in the blood declined at day 14 and after corticosteroid therapy and were undetectable at day 30. CAR T cells were able to reach the tumors and proliferate there, with peak trafficking around 1–2 weeks. Persistence was low as no CAR T cells were detected after 2 months, and lack of initial engraftment correlated with lack of detection in the tumor even early on. There was evidence of recruitment of non-transduced cells to the tumor, but T-cell infiltration was patchy. There was decreased EGFRvIII expression in five out of seven patients; one had poor engraftment, and one had stable expression. Loss of EGFRvIII has been used as a marker for treatment effect, but studies have also demonstrated that this could occur at the same rate after standard therapy and in untreated subjects [177,178]. EGFRvIII may mark a stem-cell-like population, explaining its spatial and temporal fluctuation; in some cases, it may not drive early tumorigenesis, and, therefore, targeting it alone may not lead to durable control [177]. CAR T-cell therapy resulted in compensatory resistance mechanisms evidenced by the increase in Tregs and immunosuppressive molecules IDO-1, TDO, IL-10, PD-L1, and TGF-β. Therefore, combining strategies to counteract this may be the most logical next step. As for efficacy and tumor response, no tumor shrinkage was observed in patients with multiple recurrences. There was difficulty assessing response and progression due to treatment effects and the lack of reliable imaging techniques for this treatment. Median overall survival was about 8 months, with one subject alive at 18 months with no further intervention. These were heavily pretreated patients with multifocal unmethylated tumors, factors associated with a very poor prognosis of 6 months or less [3].

Goff et al. found no objective responses based on MRI, and although it may be argued that this was confounded by treatment effect, only 3/18 patients survived longer than 12 months [58]. Furthermore, there was one treatment-related mortality due to pulmonary edema and two other treatment-related toxicities at higher doses. Lymphodepletion resulted in cytopenia requiring transfusion but no bleeding, and two patients developed VTE. CART seemed to persist for up to 3 months peripherally, but this did not correlate with survival. While the third-generation CART was developed with the goal of prolonging persistence, data have been mixed with some studies showing no survival benefit, others showing benefit, especially with low disease burden [161]. GBM patients uniquely require steroids at high doses and frequently for symptom management, and the effect is difficult to ascertain based on these small sample sizes and lack of responses, but 1-month persistence at least seemed to be equal between patients who were on steroids vs. those who were not at the start. No effect of steroid administration was observed in CD19 CART trials; however, steroids were usually used for CRS treatment rather than continually. Importantly, confirmation of EGFRvIII positivity was not required for enrollment, with an interval of months between biopsy and treatment, and confirmation in three patients showed a lack of EGFRvIII. Ongoing trials are examining alternative delivery routes, i.e., intracranially, in combination with immune checkpoint inhibition, whether as monoclonal antibodies (pembrolizumab) or by engineering them into the CAR T-cells, CAR T-cells also expressing an EGFRwt engager molecule or methods to track CAR T-cells, such as radiolabeling with Indium.

Ahmed et al. conducted a trial of HER2 CART (NCT01109095) utilizing the second-generation CAR T cells with a CD28 costimulatory domain and virus-specific T-cells (VST), with virus specificity aimed to provide further co-stimulation upon antigen presentation by APCs [81]. The use of a third-generation trastuzumab-based HER2 CART was associated with the death of one patient at doses of 10^10^ cells [179]. That study showed that CAR T-cells did not expand after infusion but could persist for up to a year at a low frequency. This is in line with VST experience in other solid tumors [180,181,182], which is in contrast to hematopoietic stem-cell transplant recipients [183,184], where the viruses are reactivated due to severe lymphodepletion. Administering viral vaccines and lymphodepleting chemotherapy may mimic this effect for solid tumor patients. An ongoing phase 1 trial (NCT03500991) of HER2-CART in pediatric patients with a variety of CNS tumors is testing locoregional delivery, either intracavitary or intraventricularly, based on the superiority of these methods in preclinical testing of CAR T therapy for medulloblastoma, ependymoma and atypical teratoid/rhabdoid tumors [185,186]. Furthermore, the CAR design was optimized for the juxta-membranous position of the HER2 epitope on the cell by altering the length of the extracellular spacer to include a medium rather than a short spacer. This is based on preclinical testing showing significant differences in cytolytic activity and tumor cell lysis. They also manufactured T-cells in a manner balancing CD4+ and CD8+ cells, with a short culture duration of <21 d, to preserve the fitness of CART cells. The initial results of three young adult patients were published and included one with a grade 3 astrocytoma and two with ependymoma [82]. CAR T-cells were not detected in CSF or peripheral blood at any point, but endogenous T-cells and inflammatory markers and cytokines, including CXCL10, which is essential for T-cell trafficking, and CCL2, which assists in homing to the tumor [187,188,189], were increased and this increase correlated with symptoms and radiographic evidence of an inflammatory response. Progressive disease was identified in two out of three patients at the end of course 2.

A trial of personalized CAR T cells against one of the common glioblastoma targets, including EGFRvIII, IL13Ra2, HER2, EphA2, CD133, and GD2, has published initial results for a cohort of three patients who received EphA2-CART administered in a single dose intravenously [87]. This study showed peripheral expansion of CART cells peaking at 7–10 days and persisting for 28 days. This was concordant with observations from systemically administered EGFRvIII-CART trials and possibly enhanced due to lymphodepletion performed as part of the protocol, as well as the engineered cells encountering the target antigen in the lung causing further expansion. This may explain why 2/3 patients experienced pulmonary edema, which might have been a result of “on-target, off-tumor” toxicity as EphA2 is not usually expressed on normal tissue except on lung epithelium and preclinical studies had suggested this as a consequence [190]. The severity of pulmonary edema also correlated with the highest level of expansion. So far, there has only been a transient tumor effect, with one patient achieving stable disease and two patients showing progressive disease on MRI by iRANO criteria.

All these studies showed that the radiological post-treatment effect can be impressive with intense inflammatory changes making it difficult to distinguish from true progression. In line with findings from multiple studies, RANO published recommendations for assessment in patients receiving immunotherapy, and these require confirmation of progression by repeat imaging 3 months later if it has been less than 6 months since the start of immunotherapy [191]. In an effort to improve imaging assessment of response, Wang et al. performed multiparametric MRI to assess 10 patients who received EGFRvIII CAR T cells [192]. This included diffusion tensor imaging (DTI), dynamic susceptibility contrast (DSC) perfusion imaging, and proton MR spectroscopy, and they used three individual parameters to calculate a progression probability. Percentage changes in any one parameter did not accurately predict progression vs. pseudoprogression; however, using this formula, they were able to accurately discern progression from pseudoprogression in all patients. This was confirmed histologically. A larger study would be needed to further validate this tool, as an accurate assessment of patients will be desperately needed as immunotherapy makes its way into the armamentarium of treatments for glioblastoma.

A report of one patient who received B7-H3 CART cells administered into the tumor cavity weekly showed an impressive radiological response after one cycle, but a very short-lived one lasting 50 days before the onset of clinical and radiological progression. The proposed mechanisms were antigen escape, as the tumor showed 50% B7-H3 expression, which was patchy, and a relatively lower CART dose. The main goal, which was to establish safety, was met as the patient only experienced headaches in relation to the infusion [92].

Finally, a study of fourth-generation, safety-designed CARs (4SCAR) targeting GD2 (NCT03170141) administered either intravenously or both intravenously and intracavitary to adult and pediatric patients with recurrent GBM was successful in demonstrating the safety of both routes as only one patient developed adverse events of a grade 3 headache and grade 2 seizures. A limitation of this study was the determination of response on MRI only 4 weeks after infusion, as this may have underestimated responses on account of pseudoprogression. Overall, 4/8 patients survived for 12 months or longer post-infusion, including 1/3 deemed to have progressed, who survived for 23 months after treatment. The shortest survival of 3 and 4 months was in patients who had a partial response and stable disease. There was evidence of antigen loss on biopsy but also remodeling of the TME, as shown by a decrease in M2-type macrophages [175]. As with all these studies, the small sample size makes it difficult to draw conclusions on which delivery route was superior.

A meta-analysis of eight [18,57,58,69,81,87,174,192] studies reported a pooled ORR of 5.1% (95% confidence interval (CI), 0.0–10.4) and a pooled median OS of 8.1 months (95% CI, 6.7–9.5). As described above, CR was only reported in studies of locally delivered IL13Ra2 CAR T [193].

### 3.4. Challenges

#### 3.4.1. Time to Manufacture

As Brown et al. found in their initial IL13-zetakine+ CAR T study, manufacturing autologous CAR T cells is a time-intensive process that takes 3–4 months. For patients with glioblastoma, for whom survival is estimated in months, this could make all the difference in preventing fatal tumor progression [18]. The process has been optimized over time but still requires at least 1–2 weeks to complete prior to infusion. This is usually due to the need for activation and expansion of T-cells ex vivo. However, it seems that this step may be omitted altogether, leading to faster production in as little as 24 h. In fact, this may result in a less differentiated phenotype with enhanced persistence, leading to better therapeutic efficacy [194].

Creating off-the-shelf or allogenic CART is another way to address this. Because of its immune-privileged status, immune rejection occurs more slowly. A study of intratumorally administered allogenic IL13Ra2 targeting CAR T cells with their glucocorticoid receptor knocked out to allow for the use of dexamethasone for rejection prevention showed feasibility and safety of this approach with only 1/6 patients having detectable antibodies and no systemic symptoms [70].

#### 3.4.2. Delivery

The optimal method is still under investigation. Regional delivery aims to bypass physical barriers that hinder T-cells from reaching the tumor, as well as avoid systemic toxicity. Multiple preclinical studies comparing locoregional delivery to systemic administration found superior results with the former [96]. Four CAR T-cell trials have tested locoregional delivery as above. It is difficult to draw conclusions on efficacy, especially in comparison to systemic delivery. Three of the trials delivered IL13Ra2 CAR T cells, which, to our knowledge, have never been clinically administered systemically. They showed an acceptable safety profile. It was evident that even direct delivery intracranially has its limitations, as intracavitary injection in one patient controlled disease locally and failed to prevent progression at distant sites. Intraventricular delivery was far superior in this specific case, with almost complete regression of all lesions. There was evidence of recruitment of endogenous immune cells and high levels of cytokines and chemokines, which the authors thought likely explained the results. In the trial of HER2-CART delivered either to the tumor or the ventricular system, no CAR T cells could be detected in the blood or the CSF, but there were markers suggestive of induction of an inflammatory response in the CNS and recruitment of T-cells. However, despite theoretically overcoming physical barriers and evidence of immune system recruitment, these studies showed a transient response in most patients, even with repeated infusions and enrichment for central memory cells in the study of IL13Ra2 CART. So it appears that even with local delivery, there are still hurdles to overcome, as described below.

T-cell trafficking and infiltration of the tumor are essential for effective treatment. It depends in large part on chemokines, which are produced by tumor cells as well as immune and endothelial cells. The main receptors on T cells are CXCR3 and CCR5, with their ligands being CXCL9 and CXCL10. Chemokine receptor mismatch has been shown to occur, compromising the successful trafficking to and infiltration of the tumor, as well as increasing the risk for systemic toxicity due to higher exposure of normal compared to malignant tissue. Optimizing the CAR to express the appropriate chemokine receptor to the tumor could improve trafficking and anti-tumor effect, as shown in a study of GD2-CAR T cells expressing CCR2b in CCL2-secreting tumors [195].

#### 3.4.3. Antigen Heterogeneity and Antigen Loss

**Improving access to antigens.** The use of tyrosine kinase inhibitors can affect the availability of tumor antigens for binding. It has been found that erlotinib and afatinib, but not lapatinib, induce dimerization of the mutant EGFRvIII receptor, increasing its stability and, thereby, possibly increasing its density or even percentage expression in the tumor [196].

As previously mentioned, single-domain VHH antibodies have the advantage of increased ability to access difficult-to-reach antigens and epitopes. Jamnani et al. engineered Jurkat T cells (an immortalized leukemia cell line) transduced with VHH-CARs targeting different epitopes [197]. The rationale was that targeting different epitopes, which is not achievable with mAbs or their derivative scFvs, would decrease antigen escape as the tumor is unlikely to downgrade all epitopes. Furthermore, this may also decrease toxicity as mAbs usually compete for the same epitope, requiring increasing doses and a higher risk of side effects. These oligoclonal Jurkat cells indeed showed superior expansion and function when compared to non-oligoclonal cells. However, this was performed in vitro and using Jurkat cells. More studies are needed to confirm the utility of this design.

**Targeting Multiple Antigens.** Bispecific CARs targeting different antigen combinations have been tested in preclinical studies of GBM. A tandem CAR (TanCAR) targeting HER2 and IL13Ra2 showed the ability to induce heterodimerization of both targets, resulting in super-additive activation without increased exhaustion in comparison to bispecific CART (expressing separate HER2-CAR and IL13Ra2 CAR) or uni-specific CART and ultimately prolonged survival with doubling of progression-free survival. Despite increased activation when encountering double-positive cells, exhaustion levels did not seem to increase, the reason for which is not clear. The therapeutic effect was more sustained at higher doses. With the administration of lower TanCAR doses, recurrent tumors were double-negative, suggesting that long-term suppression could be improved by broadening the targeting to include other elements of the TME that sustain tumor cells despite initial therapy success [198]. The importance of determining an effective dose was also demonstrated by a study on TanCAR targeting CD70 and B7-H3, both of which are expressed in glioblastoma and have been studied individually, where there was incomplete eradication and, ultimately, antigen loss [199]. Further studies testing the TanCAR design were conducted for EGFRvIII and IL13Ra2, as well as EphA2 and IL13Ra2, also showing superior efficacy in vivo [200,201]. Building on their work with HER2/IL13Ra TanCAR and in an attempt to address the limitations mentioned, Bielamowicz et al. designed trivalent CAR T with the addition of EphA2 and the intent to capture 95% or more of the tumor population. The superiority of this design was demonstrated against the best univalent and bivalent CAR T designed for the respective patient-derived xenograft models [202]. This addressed the problem of heterogeneity, but this is only one aspect of designing therapy for clinical use, and other important aspects, such as persistence, remain. Most importantly, when targeting multiple antigens expressed systemically, there is concern for significant toxicity. Another problem to keep in mind when engineering TanCARs is the large size of the vectors required for their genetic engineering when using scFvs, which may lead to decreased viral transduction efficacy [203]. Ahn et al. used nanobodies to construct bispecific CARs targeting HER2 and EGFR or biparatopic EGFR CARs and demonstrated their efficacy both in vitro and in vivo [204]. This is promising and could be expanded to target even more antigens using one construct.

SynNotch CAR T is a design that recapitulates the concept of CAR secreting a bispecific antibody but only under certain conditions to avoid off-tumor killing while targeting more ubiquitous antigens. The synNotch receptor recognizes an antigen that is highly tumor-specific but not homogeneously expressed, such as EGFRvIII, and subsequently activates a transcriptional program leading to the expression of CAR geared toward a different antigen that is more widely expressed. This IF-THEN mechanism ensures a limited effect within tumor tissue, as killing through CAR is activated only after cells have been primed. This was tested in a preclinical study using EGFRvIII as the priming antigen and Tandem CAR directed against both EphA2 and IL13Ra2. It showed that EGFRvIII SynNotch-EphA2/IL13Ra2 CAR T cells could effectively eradicate EGFRvIII negative cells even with EGFRvIII expression as low as 10%. In vivo, tumor control was equal between 50% and 100% EGFRvIII+ tumors, showing that this strategy can effectively overcome antigen heterogeneity. It also confirmed that no killing occurred in the absence of the priming antigen, evidenced by unchecked growth of EGFRvIII- tumors implanted in the flank despite significant control of intracranial EGFRvIII+ tumors in the same mice [205]. Only 30% of patients with GBM harbor the EGFRvIII mutation, but fortunately, EphA2 and IL13Ra2 are not expressed in normal brain tissue, making it sufficient to target a CNS-specific and not necessarily a tumor-specific antigen. Mice treated with MOG SynNotch-EphA2/IL13Ra2 CAR T cells again showed superior tumor control and survival with no killing detected outside of the tumor in normal brain tissue [205].

There remains a subset of GBM tumors that may not express any of the described antigens, especially recurrent GBM and especially those who received prior immunotherapy targeting those antigens, as demonstrated by preclinical and clinical data so far. Chlorotoxin (CLTX) is a peptide derived from scorpion venom that was found to bind specifically to glioblastoma cells and minimally to normal brain or tissue. A study of CLTX-CART attempted to exploit this for T-cell retargeting. They found that CLTX-CAR T binds to 80% or more of tumor cells in 13/15 patient tumor samples, with the other two displaying 40% binding, regardless of HER2, IL13Ra2, or EGFR expression levels. This appears to be mainly mediated by the presence of membrane-associated matrix metalloproteinase 2 (MMP2). In recurrent tumors, binding was still observed, suggesting that antigen escape was not the mechanism of recurrence; rather, there was upregulation of immunosuppressive factors, specifically PD-L1, on tumor cells and decreased GzmB positivity of CAR T cells. Toxicity studies showed accumulation in the lungs after systemic administration but no GzmB production and no observed adverse effects [96]. An ongoing phase 1 trial is testing the same CLTX-CAR T in MMP2-positive glioblastoma patients (NCT04214392).

Another way to deal with antigen-negative tumor cells, which could contribute to antigen escape, is to sensitize them to the bystander-killing effect of CAR T cells. This was achieved in preclinical models by combining CAR T with an inhibitor of apoptosis protein (IAP) antagonist such as birinapant. This is promising, but it should be noted that not all tumors are sensitive to its effect, and combination with a resistance-modifying agent may be necessary, the design of which in itself is no easy feat [206].

#### 3.4.4. Persistence, T-Cell Exhaustion, and the Microenvironment

**Pre-conditioning.** Preparing the environment prior to administration of therapy to make way for CAR T cells to maximally exert their effect can take multiple forms.

***Preparing the patient.***

Lymphodepletion (LD) is usually achieved by administering cyclophosphamide or, fludarabine, or both and is a necessary step in hematological therapies. This can enhance CAR T therapy by killing tumor cells, removing IL-7 and IL-15 competition, making them more available to CAR T cells in the absence of endogenous lymphocytes, and, therefore, improving CAR T-cell proliferation and persistence. It may also eliminate Tregs and myelocytic-derived suppressor cells (MDSC). Glioblastoma patients receive temozolomide as part of standard therapy, and this can cause lymphodepletion to varying degrees. However, a study on a murine model revealed that standard dosing is not sufficient to facilitate CAR T-cell proliferation as desired, and a dose-intense regimen is required. Not only that, but they showed that systemically delivered EGFRvIII CAR T alone completely failed at controlling tumors without prior lymphodepletion, which was consistent with previous experiments. The mechanism appears to be an overall increase in CAR T-cell numbers in the brain and a higher ratio of CAR T to Tregs [207]. Many preclinical studies in GBM showed a significant contribution and a better outcome by incorporating LD [49]. This would have to be further validated as only two of the nine available studies in GBM patients included lymphodepletion as part of their protocol, and although results were mixed, impressive responses were obtained in studies that did not include lymphodepletion.

***Preparing the microenvironment.***

Oncolytic viruses can also be utilized in this manner to prime the tumor prior to delivery of CART. A CXCL11-armed oncolytic adenovirus (oAd-CXCL11) was administered in a GBM model prior to B7-H3 CART. CXCL11 is another highly important chemokine in T-cell recruitment to the tumor, which binds to the CXCR3 receptor. Administering oAd-CXCL11 not only resulted in increased T-cell infiltration but also remodeled the TME by increasing numbers of NK and M1 macrophages and decreasing MDSCs, Tregs, and M2 macrophages. A previous study of CAd-Trio with CAR T showed only transient responses in immunodeficient models [137]. In this study, combination therapy was indeed superior to either monotherapy and prolonged survival in immunocompetent GBM models, highlighting the importance of the endogenous immune system and the need to recapitulate that in preclinical studies [208].

Normalizing TME vasculature is another way to increase the probability of successful CART infiltration and efficacy. Multiple factors have been implicated in tumor angiogenesis, the most well-known of which is vascular endothelial growth factor (VEGF) [209]. The use of anti-VEGF prior to EGFRvIII-CART improved infiltration as well as distribution within the tumor and significantly prolonged survival of mice in comparison to EGFRvIII alone [52]. Phosphoglycerate dehydrogenase (PHGDH) is a stress-induced enzyme expressed in endothelial cells that drives aberrant vessel sprouting in GBM. PDGDH-EC inhibition in combination with EGFRvIII-CART showed pruning of abnormal vasculature and improved infiltration and activation of T-cells, correlating with significantly improved survival in murine models with some complete responses, compared to either monotherapy alone or pretreatment with VEGFR2 inhibition [53]. A similar improvement of EGFRvIII-CART was achieved by targeting yet another regulator of abnormal angiogenesis, p21-activated kinase 4 (PAK4) [51].

***Persistence: Exploiting cytokines.***

Cytokines orchestrate almost every aspect of T-cell and other immune cell functions, and their balance can alter the entire microenvironment, influencing whether this would potentiate or suppress cytotoxic and other tumor functions. Their use in cancer therapy, specifically CART, has been extensively studied [163,210].

In a murine model, a single dose of IL-12 delivered locally to the tumor in addition to EGFRvIII-CART showed improvement over EGFRvIII alone. This was found to be related to decreased T-cell exhaustion, increased inflammatory CD4+ cells, decreased Tregs, and modulation of the myeloid compartment toward more reactive phenotypes. Upregulation of PD-L1 was observed on MDCs, suggesting a possible benefit of ICI combination therapy. IL-12 was not detected in the serum, and only IFN-y and CXCL9 were, suggesting the safety of this approach [211].

Engineering CARs to co-express cytokines, whether by leading to their local secretion, such as in TRUCKs, or by inducing their signaling, such as in fifth-generation CARs, is an elegant way of delivering their benefits while avoiding their systemic effects.

Interleukin-7 (IL-7) has been found to improve T-cell expansion, increase memory cells, increase IFN-y production, and decrease Tregs in preclinical GBM studies. Recombinant IL-7 is being tested for GBM in a phase 2 trial (NCT03687957). A preclinical study on immunocompetent mice with heterogeneous, 50% EGFRvIII GBMs preconditioned with non-lymphodepleting irradiation found that administration of EGFRvIII-CART expressing either IL7 or IL7 and Flt3L (Fms-like tyrosine kinase receptor 3 ligand), which is a cytokine essential for dendritic cell (DC) function, significantly increased the abundance of CAR T cells and CD8 cells and improved survival compared to CAR alone. The effects of IL7 seemed to dominate, although IL7 and Flt3L increased DCs in the tumor. There was no difference in exhaustion levels as those remained low across all groups, which the authors attributed to the third-generation design using both CD28 and 4-1BB [212]. In a different approach, IL-7-loaded oncolytic adenovirus in conjunction with B7H3-CART cells were tested in GBM mouse models and showed improved proliferation and persistence of CAR T cells, with a three-fold increase in number of cells, as well as improved tumor control in advanced tumors compared to CAR T cell alone. CAR T alone had a transient effect. An increase in PD-1 and LAG-3 positive TILs was observed. The authors regarded it as a marker of activation as well as exhaustion [213]. An alternative to IL-7 secretion is including a constitutively active IL-7 receptor in the CAR design, which activates STAT5 signaling and avoids possible toxicity due to accumulation of IL-7. In a study of CART with an active IL-7 receptor (C7R), it was found that, while there was no significant difference in potency of cytotoxicity initially, there was a clear advantage to GD2-CAR.C7R cells over GD2-CAR cells upon rechallenging with the antigen. This was due to enhanced proliferation and resistance to apoptosis even up to the third tumor rechallenge. There was also a clear advantage with longer persistence but without induction of proliferation in the absence of antigen or autonomous proliferation. This translated into tumor eradication and maintained response in comparison to only a 1-week survival advantage with GD2-CART alone in the neuroblastoma model [214]. The experiment was replicable with EphA2-CART in a glioblastoma model. Dual-target, IL7Ra fourth-generation Tris-CAR-T cells are being tested in a phase 1 trial (NCT05577091).

***Targeting the TME.***

TGF-B is a known major barrier to multiple forms of immunotherapy as it is a powerful immunosuppressive effector in the TME. However, TGF-B inhibitors such as galunisertib have had no clear benefit and failed to improve survival when added to standard temozolomide and radiotherapy [215] or in comparison to or in combination with lomustine [216]. When it comes to cellular therapies, however, addressing TGF-mediated immunosuppression becomes more urgent as it has known effects on T-cell functions that would impair CART or NK. Rather than inhibiting TGF-B itself, CAR T cells could be engineered to become insensitive to it. This can be achieved by using CRISPR/Cas-9 to knock out the TGF-B receptor or to overexpress dominant-negative TGFBRII (TGFBRDN). Preclinical studies conducted as part of a phase 1 trial of PSMA-CAR T cells favored TGFBRDN due to superior T-cell proliferation [217]. However, there was an increase in suppression factors such as IDO1, CD40, Tim-3, and PD-L2 in tumor samples of treated patients. Furthermore, there was no uniform tumor infiltration by T cells. Overall, this trial showed preliminary evidence of antitumor effect in heavily pretreated patients. DnTGFBRII was found to decrease the proportion of Tregs in tumor tissue, increase differentiation of T cells into effector cells, and induce effector molecule production such as IFN-y, granzyme B, and perforin. One possible mechanism of TGF suppression of T-cells is the inhibition of their differentiation into effector phenotypes, and removal of this inhibition enables better tumor eradication. However, as discussed, a more differentiated effector state may not be optimal for long-term control of disease by CART cells, and so, the implications on long-term outcomes need further study.

Targeting the immunosuppressive cells in the TME is a promising strategy, as described in the previous section, with CAFs and macrophages, but it is likely insufficient on its own without targeting tumor cells. Myeloid-derived suppressor cells (MDSC), a main player in immunosuppression, were found to express IL15Ra receptor, and this was utilized to create IL13Ra2-CART which either secreted IL15 or expressed it as part of the targeting domain of the CAR. The latter design was found to be more effective. Indeed, this resulted in the depletion of MDSC and immunosuppressive factors such as TGF-B, Arginase 1, and IL10, increased CD8+ T cells, B cells, and NK cells, and prolonged survival in syngeneic GBM models [68].

Toosendanin is a small molecule that is capable of reprogramming macrophages and was found to improve CAR T-cell infiltration, inhibit exhaustion, and ultimately positively impact survival in GBM models when administered prior to EGFRvIII-CART. Furthermore, combining TSN-sensitized GBM to ICIs and the combination of TSN, ICI, and CART induced complete and durable responses in 2/3 of the mouse models [54].

Non-cellular factors such as the extracellular matrix itself are important to consider as they form a further layer of immunosuppression and prevent effective trafficking and infiltration. An interesting approach tested in other solid tumors is designing a heparinase-secreting CAR T-cells that could degrade heparin sulfate proteoglycan (HSPG) in the ECM. Apart from T cells, CAR-macrophages designed to trigger CD147 signaling and metalloproteinase secretion upon encountering their CAR target antigen showed effective tumor control and significantly increased T-cell infiltration into tumors [173,218].

Nanobody-based CARs targeting the TME through PD-L1 or EIIIB+ fibronectin splice variant, a crucial component of the tumor stroma, has proven beneficial in delaying tumor growth in animal models [219]. Furthermore, CAR T cells targeting elements of the TME, such as PD-L1, which secrete VHH, showed the potential and great flexibility of nanobodies. PD-L1-CART secreting VHH or VHH-Fc fusions against CD47 in an effort to engage the innate immune system showed prolonged survival in immunocompetent animal models. This is an elegant solution to contain both the ICI and effects of CD47 antagonism within the tumor. These VHH-secreting CAR T-cells also demonstrated decreased exhaustion, potentially solving yet another problem with CART, which is discussed below [220].

***T-cell Exhaustion.***

An exhausted T-cell harbors the exhaustion markers, which most prominently include the immune checkpoints PD-1, Tim-3, and LAG-3, among others. These cells lose proliferative and cytotoxic capability [221].

One hypothesized mechanism of CAR T-cell exhaustion is tonic signaling in constitutively expressed CARs. Prolonged exposure to antigen is known to lead to T-cell exhaustion, but in CART, this appears to be antigen-independent and the result of self-association and clustering of receptors causing excessive activation and, therefore, exhaustion. It was also found that 4-1BB signaling improves the exhaustion profile, as opposed to CD28 [222]. Interestingly, SynNotch CART showed decreased signs of tonic signaling and a tendency for less differentiation, with an increase in stem-cell-like phenotypes, leading to longer persistence [205]. This is in line with the finding that transiently “turning-off” CAR T cells could ameliorate exhaustion [223].

It is interesting to note that in the study of CLTX-CART, the type and length of the spacer also influenced exhaustion. Although both CLTX-CD8h-28ζ and CLTX-EQ-28ζ CART showed increased PD-1, this was associated with upregulation of other markers of exhaustion and less durable function with CD8h [96].

Metabolism is a unique challenge for cells as opposed to antibodies but applies in either case as cells are the effectors, and in the case of CAR-T, the advantage lies in the ability to engineer mechanisms of resistance to this environment into them. CAR T cells can be engineered to resist immunosuppressive factors resulting from hypoxia, such as adenosine, through adenosine A2A receptor knockout or antagonism [224,225,226], or effects of reactive oxygen species (ROS), through the expression of catalase to neutralize H_2_O_2_ (CAR-CAT) [227].

Furthermore, the programs leading to exhaustion themselves could be overcome by blocking transcriptional factors of exhaustion, such as NR4A, TOX, and TOX2 [228].

***Immune checkpoint inhibition.***

Upregulation of immune checkpoints in response to CART has been an almost universal observation in studies and provides a therapeutic opportunity for incorporating ICI in CAR T for GBM.

It is important to note that different antigen-targeting CARTs are associated with the upregulation of different immune checkpoint profiles, as demonstrated by the improved efficacy of IL13Ra2-CART in combination with CTLA-4, while EGFRvIII-CART was improved when combined with PD-1 or Tim-3 [229]. Despite the efficacy demonstrated by preclinical studies [229,230], there remains the issue of immune-related adverse events (iRAE) associated with systemic ICIs [231]. CAR T-cells secreting an ICI could eliminate systemic toxicity and improve the delivery of the ICI to the tumor. The study of IL13Ra2-CART found that this led to less stimulation and cytokine secretion when a PD-1 minibody was included, while there was an added benefit with CTLA-4 minibody secretion [229].

Beyond ICIs, CAR T-cell intrinsic PD-1 blockade has been achieved by either knocking out the PD-1 receptor, as was performed in EGFRvIII-CART cells for glioma [56], or by overexpressing a dominant negative receptor that lacks an intracellular signaling domain [55]. Similar to the concept of SMiTE [135], a chimeric switch receptor binds to PD-L1 but has trans-membranous and intracellular CD28 domains that switch the signal from an inhibitory to a costimulatory one [232,233]. The tandem CAR design can also be used to target PD-L1 and a tumor-associated antigen rather than two antigens [234].

Two ongoing phase 1 trials are testing the combination of IL13Ra2-CART with Ipilimumab (CTLA-4i)/Nivolumab (PD-1i) or Nivolumab alone (NCT04003649) and EGFRvIII-CART with Pembrolizumab (PD-1i) (NCT03726515).

#### 3.4.5. Toxicity

CAR T-cell therapy is associated with unique and possibly life-threatening adverse effects. Cytokine release syndrome (CRS) is the most common, occurring at rates of approximately 40–90% in lymphoma and leukemia trials. It usually presents within the first week with fever as the first symptom, with or without other constitutional symptoms, which could quickly evolve into severe hypotension and hypoxia. It is associated with high levels of serum IL-6 and is usually treated with a combination of steroids, tocilizumab, and supportive care. Immune-effector-cell Associated Neurologic Syndrome (ICANS) is the second most common, with rates ranging from 23–67% in lymphoma and leukemia. It usually starts with confusion, progressing to expressive aphasia, which is a distinct feature, later developing into global aphasia with other focal neurological deficits, such as weakness, seizures, altered level of consciousness, and cerebral edema in the most severe cases. Peak incidence is within the first 2 weeks, closely following CRS, but it can occur as late as 3–4 weeks. In most cases, symptoms are fully reversible with the administration of dexamethasone [235].

The meta-analysis of eight of the CAR T-cell therapy studies in glioblastoma found that out of 63 patients, 9.5% developed CRS, and 25.4% experienced neurological side effects. Based on this data, CRS and neurological toxicity appear to be much less frequent in GBM compared to hematological malignancies. Neurological symptoms are commonly a result of the disease itself or even the treatment effect, and it is difficult to attribute them with certainty to the treatment. Furthermore, reported neurological adverse events were mild to moderate and transient [193].

Innovative CAR designs have been developed to address toxicity by a variety of mechanisms.

Simply altering the hinge domain may decrease CAR sensitivity to low expression of the antigen, such as those expressed in healthy tissue. This was demonstrated for EGFR and HER2 targeting CARs. This strategy also depends on antigen location, as truncation of the hinge domain for EGFRvIII CARs did not decrease sensitivity, which is thought to be due to the distal location of EGFRvIII from the membrane as opposed to HER2 [236].

We have mentioned several methods which, when targeting antigens present on healthy tissue, could limit killing to the tumor environment by requiring the presence of a tumor-specific antigen such as SynNotch CAR and CAR-secreting BiTEs [31,205,237].

Hypoxia-inducible CARs (HiCAR) capitalize on the tumor’s hypoxic environment by including a hypoxia-inducible factor 1a (HIF1a) oxygen-dependent degradation domain to ensure the CARs expression only under these conditions, driving CAR T cells away from normal tissue due to their oxygen sensitivity. This has been tested with multiple CARs, including HER2, for solid tumors [238].

Signal neutralization by an inhibitable protease (SNIP) CARs is, as the name suggests, designed to cut the CAR upon withdrawal of the small molecule drug. The dose could be titrated to achieve an optimal balance between toxicity and antitumor effect. These CARs were tested in multiple tumor models and were further shown to have superior efficacy and better safety compared to constitutive CARs, likely owing to the rest effect and decreased CAR T-cell exhaustion [239].

CAR T-cells can be transduced with an inducible caspase9 (iC9) suicide gene. In the study of GD2-CAR.C7R, this did not result in loss of anti-tumor efficacy, and administration of chemical inducer of dimerization AP20187 (CID) immediately resulted in loss of CAR T cells in vivo [214].

The field of CAR engineering has advanced at an exponential pace, and many other designs now exist that could add the ability to control the CAR’s activity, thereby improving safety and efficacy. Inhibitory CAR T cells (iCARs) have a second CAR that recognizes an antigen expressed on normal but not malignant tissue, which is connected to an intracellular domain similar to that of immune checkpoints and which could inhibit cytotoxic function once those are recognized. Other designs have a switch-on or off mechanism, similar to SNIP CARs. Split CARs can be designed to either be constitutively split into two components, which require a dimerizing agent to become a functioning CAR, or the reverse design with the drug able to disassemble the CAR when necessary. Double-arm CARs contain a signal CAR that recognizes a TAA and a scissor CAR attached to it, which recognizes a normal tissue antigen, which, when recognized, could cause the scissor CAR to cut the signal CAR. Furthermore, universal CARs (UniCAR) include a binding moiety that can bind multiple different antigen-targeting modules for easy retargeting and control. Many others exist, but a discussion of these designs is beyond the scope of this review, and multiple excellent reviews discuss them at length [166,240,241].

## 4. Summary

**Figure 4:**
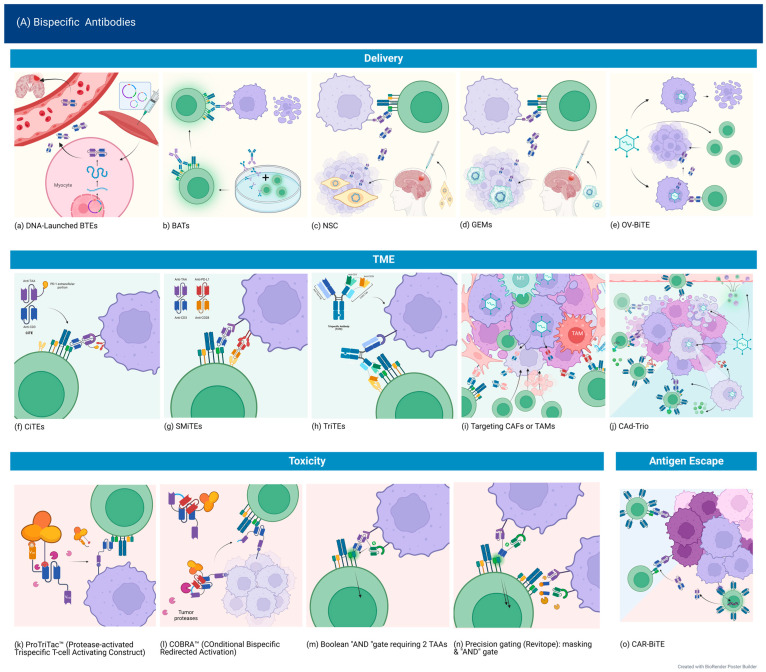

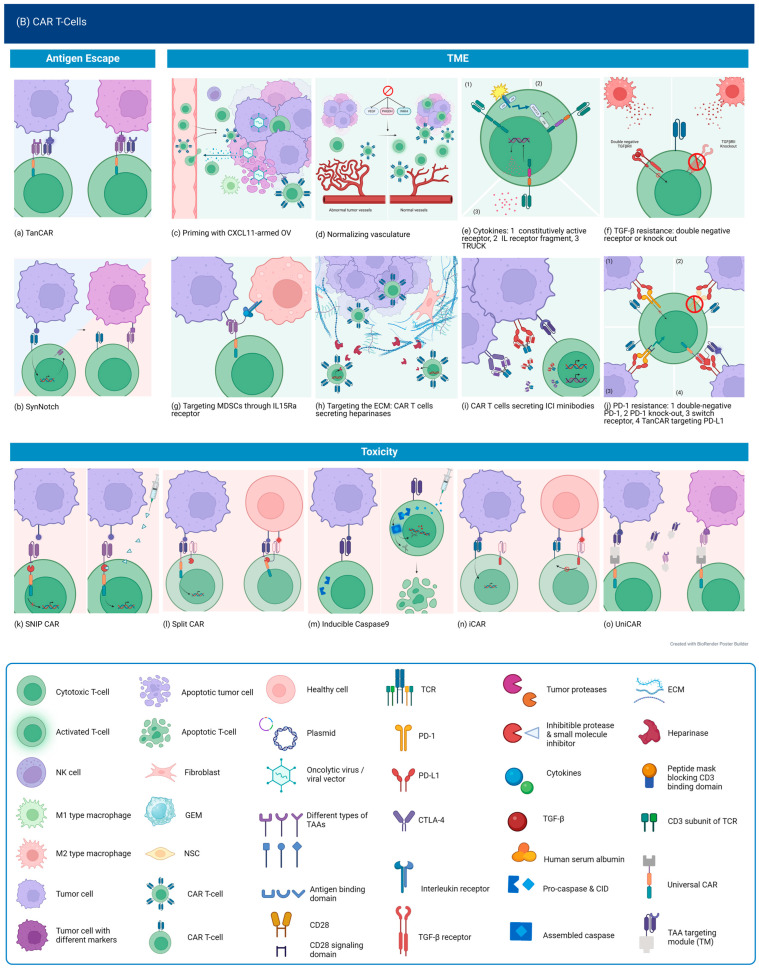
Novel approaches addressing challenges of bispecific antibodies and CAR T-cell use in the treatment of solid tumors. Created with BioRender.com (accessed on 12 September 2023).

Bispecific T-cell engagers and CAR T-cell therapies have recently emerged as an exciting avenue in cancer treatment, and their use is being explored for the treatment of glioblastoma. Much research has been conducted, but there remains a significant amount of work before these therapies can be incorporated into clinical practice. Bispecific antibodies have promising preclinical data for efficacy in glioblastoma, but many challenges remain as above. Refining the delivery mechanism to bypass the BBB and increase half-life, such as by local secretion by the resident or immune cells or even oncolytic viruses, targeting multiple antigens using designs such as trispecific antibodies, using conditional designs which are contingent on the presence of multiple tumor markers to enhance safety, and combining it with other immunotherapies such as CAR T cells and oncolytic viruses are promising methods under study. CAR T-cells have more clinical data available, although it is all from small studies with mixed methodologies and mixed results. Again, the challenges include methods of delivery, and whether intracranially or systemically remains an important question. Antigen escape can again be addressed by increasing the number of targets or combining immunotherapies such as in BiTE-secreting CAR T-cells. CAR T-cells are live drugs and provide many opportunities for flexible designs, although T-cell persistence, trafficking, and exhaustion are especially problematic. They also require a costimulatory signal, and the choice seems to alter function significantly. Utilizing next-generation designs with cytokine or chemokine activity and engineering CARs for intermittent rather than constitutive activity may address these issues. CARs targeting elements of the tumor microenvironment or combined with immune check-point inhibitors or oncolytic viruses may be able to overcome this particular hurdle in effective treatment. Toxicity is a big concern, but designs such as SynNotch, universal CARs, inhibitory CARs, and many others mentioned seem to have great potential to minimize these. Figure 4 includes some of the most notable methods aimed at addressing challenges for the use of T-cell-engaging therapies. Ultimately, more data is needed to determine if the immense cost of these treatments is justified, especially with their personalized nature requiring comprehensive genetic testing for each patient and their toxicity, which further adds to the cost. But if these new designs bear fruit and are clinically successful in achieving similar results as those in hematological malignancy for tumors such as glioblastoma, it certainly would be the case.

## Figures and Tables

**Table 1:** Bispecific antibody and CAR T-cell targets investigated in GBM.

Antigen	Properties	BsAbs	CART	
			Preclinical	Clinical
EGFR	EGFR amplification is the most common genetic alteration in GBM, present in about 50% of tumors.	BATs: Huang et al., 2022 [34] CAR.BiTE: Choi et al., 2019 [31]	Jiang et al., 2018 [35] Ravanpay et al., 2019 [36] Xia et al., 2021 [37] Thokala et al., 2021 [38]	None
EGFRvIII	A variant resulting from a mutation that leads to deletion of exon 2–7 and a subsequently dysfunctional, constitutively active receptor promoting tumorigenesis; Associated with poor prognosis; Unclear if it is a late or early marker of tumorigenesis; GBM expression: occurs most commonly in the setting of EGFR amplification [39]. Present in about 30% of GBM, but expression is not homogeneous and fluctuates; Normal tissue expression: none.	Clinical: AMG 596: NCT03296696 BRiTE: NCT04903795 RO7428731: NCT05187624 Preclinical: Choi et al., 2013 [40] Ellwanger et al., 2017 [12] Gedeon et al., 2018 [41] Sun et al., 2021 [24] Iurlaro et al., 2022 [42] DICE: Park et al., 2020 [43] dBTE: Park et al., 2023 [44] GEMs: Gardell et al., 2020 [45]	Bullain et al., 2009 [46] Ohno et al., 2010 [47] Ohno et al., 2013 [48] Sampson et al., 2014 [49] Johnson et al., 2015 [50] CAR.BiTE: Choi et al., 2019 [31] Anti-angiogenic: Ma et al., 2021 [51], Don et al., 2023 [52], Zhang et al., 2023 [53], Yang et al., 2023 [54] PD1: Chen et al., 2016 [55], Zhu et al., 2020 [56]	O’Rourke et al., 2017: [57] NCT02209376 Goff et al., 2019: [58] NCT01454596 NCT03726515 NCT05063682 NCT03283631 NCT02844062 NCT02664363 NCT05660369 NCT05024175 NCT05802693
IL13Rα2	Decoy receptor for IL-13 and IL-4. May increase tumor proliferation through the PI3K-AKT pathway, promotion of TGF-β secretion, and immunosuppressive gene expression. Associated with mesenchymal subtype and poor prognosis; GBM expression: overexpressed in 50% of GBM [59], by stem-cell like and more differentiated cells [60], and in 76% of IDH-WT and TERTp-mutant GBMs; Normal tissue expression: minimal to none in brain tissue [61,62,63,64].	dBTE: Bhojnagarwala et al., 2022 [65] NSC: Pituch et al., 2021 [66]	Kong et al., 2012 [67] IL15: Zannikou et al., 2023 [68]	Brown et al., 2015: [18] NCT00730613 Brown et al., 2016: [69] NCT02208362 Brown et al., 2022: [70] NCT01082926 NCT02208362 NCT04661384 NCT05540873 With ICI: NCT04003649
Fn14	A transmembrane protein belonging to the tumor necrosis factor receptor family. The sole receptor of Tumor necrosis factor-like weak inducer of apoptosis (TWEAK); Role in tumor progression, migration, and survival through resistance to cytotoxic therapy [71]. Correlated with worse outcomes and shorter survival; GBM expression: 5-fold vs. normal brain in 68% on both GBM cells and endothelial cells (ECs). Higher in IDH-wt and recurrent; Normal tissue: minimal to none in normal brain [72].	Li et al., 2021 [73]	Li et al., 2021 [73]	None
CD133	GSC marker but also expressed on various normal stem cells, including hematopoietic cells at lower levels. Targeting may have a significant effect on hematopoiesis at high doses [74]; Contributes to tumorigenesis and chemo- and radio-resistance through CSC self-renewal ability.	DATE: Vora et al., 2020 [74]	DATE: Vora et al., 2020 [74]	NCT05577091
CA9	Hypoxia-induced cell surface enzyme that plays an important role in maintenance of stem cell survival and therapeutic resistance [75,76]; Its expression in normal tissue is limited to gastrointestinal cells.	DATE: Tatari et al., 2020 [77]	None	None
HER2	Transmembrane glycoprotein with tyrosine-specific kinase activity. Known oncogenic protein; GBM expression: over-expressed in 76–80% [78]; Normal tissue: expressed at low levels in ~60% of normal brain.	NKG2D CAR-NK: Zhang et al., 2021 [79] dBTE: Park et al., 2023 [44]	Ahmed et al., 2010 [80]	Ahmed et al., 2017: [81] NCT01109095 Vitanza et al., 2021: [82] NCT03500991 NCT03389230
EphA2	Eph receptors bind to their cell-bound ephrin ligands and play a key role in cell adhesion, migration, and proliferation; Present in high levels on most tissues during development, much less on adult tissue, except on tumors, especially CSCs; EphA3: present in ~40% of gliomas, especially mesenchymal GBM, and correlating with worse prognosis. Expression ranges from universal to only a subset of the tumor cells. More highly expressed on GSCs [83]; EphA2 and A3 enriched in recurrent GBM and co-expressed on GSCs. Knockdown of both EphA2 and 3 prevents tumor formation.	EphA2 and A3: Qazi et al., 2018 [84] Engager T cell: [85]	Chow et al., 2013 [86]	Lin et al., 2021: [87] NCT03423992
B7-H3	Immune checkpoint molecule; GBM expression: ~70% of GBMs, enriched in CSCs; Normal tissue: very low/none [88].	None	Tang et al., 2019 [89] Nehama et al., 2019 [90] Haydar et al., 2021 [91]	Tang et al., 2021 [92] NCT04385173 NCT04077866
GD2	GBM expression: ~80%; Normal tissue: low levels in CNS, sensory nerves, melanocytes, lymphocytes, and mesenchymal stem cells.	None	Prapa et al., 2021 [93] Gargett et al. [94]	NCT03170141 NCT04099797
Chlorotoxin/MMP2	CLTX: peptide derived from scorpion venom found to bind to gliomas at a very high frequency; MMP2 promotes invasion and metastasis; GBM expression: 76% [95]; Normal tissue: 12.5% on normal brain.	None	Wang et al., 2020 [96]	NCT04214392
CD147	Induces fibroblasts to secrete metalloproteinases, promoting tumor invasion; GBM expression: strongly–moderately positive in 54% of GBM’s, at much higher levels than normal brain; Normal tissue: low levels in multiple.	None	None	NCT04045847
CD70	Surface ligand to CD27, a costimulatory molecule; Mediates tumor escape through immune cell apoptosis, promotes macrophage infiltration and metastasis; GBM expression: 35% of primary, 69% of recurrent GBMs. Heterogenous ~70%; Normal tissue: none.	None	Jin et al., 2018 [97]	NCT05353530
NKG2D	A receptor present on natural killer cells and other lymphocytes which recognizes NKG2D ligands such as MHC class I-related molecules MICA and MICB on tumor cells and leads to their lysis; NKG2D-L expression can be induced by chemo and RT [98].	+CAR NK: Zhang et al., 2021 [79]	Weiss et al., 2018 [99] Yang et al., 2019 [100] Meister et al., 2022 [101]	NCT05131763 NCT04717999
Podoplanin	Transmembrane glycoprotein on endothelium; GBM expression: 16-fold that of normal brain in 30%, especially mesenchymal.	None	Shiina et al., 2016 [102]	None

NKG2D: Natural Killer Group 2D.

**Table 2:** Completed and ongoing * trials of bispecific Tcell engagers and CAR T-cell therapy in GBM.

	Population		Intervention	Outcome	Toxicity	Other Findings
IL13Ra2						
Brown et al., 2015 [18] (NCT00730613)	3 patients with recurrent, unifocal, resectable grade 3 or 4 glioma		CAR: 1st generation (IL13-zetakine) Route: Repeated IC injection following resection Dosing: 10^7^ to 10^8^ in 12 doses over 5 weeks LD: no	Mean survival 11 mo Longest survival 14 mo	Grade 3 neurological A/E at 10^8^ 1 pt with 2 headache episodes 1 pt with tongue deviation and gait disturbance	Transient MRI worsening correlated with highest antigen expression Transient response Antigen loss Time to manufacture limited enrollment Persistence: up to 14 weeks
Brown et al., 2016 [69] (NCT02208362)	1 patient with MGMT unmethylated GBM and multifocal/LM recurrence after standard Rx and Infigratinib trial		CAR: 2nd generation, 4-1BB, enriched for T_CM_ Route: IC + IVT Dosing: 5 IC infusions of 2 mil then 10 mil cells followed by 10 additional IVT treatments for recurrence LD: no	Stability of treated tumor cavity Decrease of 77–100% in lesions’ size after 5th IVT Rx Response maintained for 7.5 months from the first Rx	Grade 2, within 72 h: headache, fatigue, myalgia, olfactory aura	Antigen loss CAR T detected in CSF at all points but not in PB Decreased number of CAR T cells with decreasing tumor burden Increase in endogenous cells; recruitment of immune system Cytokines increase by 10-fold correlating with A/E.
Brown et al., 2022 [70] (NCT01082926)	6 patients with non-resectable recurrent grade 3 or 4 on steroids		CAR: Glucocorticoid receptor negative allogenic IL13 zetakine CTLs infused Route: IC Dosing: 4 cycles, twice weekly × 2 weeks, followed by IC IL-12 d2-5 and then d1-5 LD: no	Median OS 2.9 mo Longest 11.5 mo	Grade 1: injection site reaction, fever Grade 1/2: H/A, confusion, fatigue, tachycardia, distant stroke 2 weeks later	Manufacturing: 5 months Use of dexamethasone did not decrease CAR cytotoxicity but may have abrogated endogenous response
Ongoing	Criteria		Intervention	Status	Start	Completion
NCT02208362 City of Hope	82 pts with Recurrent/Refractory any grade glioma		CAR: Autologous IL13(EQ)BBzeta/CD19t+ TCM-enriched or naïve/memory (TN/MEM) T Cells Route: ITu, IC, IVT, or 2 locations		May 2018	June 2023
NCT04661384 City of Hope	Leptomeningeal mets from Ependymoma, Glioblastoma, or Medulloblastoma		CAR: Autologous IL13Ralpha2-specific Hinge-optimized 41BB-co-stimulatory Route: IVT q1 week × 4	Recruiting	December 2020	November 2025
NCT04003649 City of Hope	Recurrent glioblastoma		CAR: Autologous IL13Ralpha2-specific Hinge-optimized 4-1BB-co-stimulatory With or without nivolumab + ipilimumab or nivolumab alone	Recruiting	December 2019	November 2023
NCT05540873 (MAGIC-I) CellabMED	Recurrent or refractory grade 3 or 4 glioma		CAR: IL13Ra2 CART Route: IV	Recruiting	July 2022	April 2024
EGFRvIII						
O’Rourke et al., 2017 [57] (NCT02209376) Terminated to pursue combination Rx	10 pts with recurrent GBM		CAR: CART-EGFRvIII, 2nd-generation, 4-1BB costimulatory domain Route: IV, single infusion LD: no	Median OS 10 mo	No DLT, no CRS 3 pt received Siltuximab for neuro Sx	Antigen loss, heterogeneity Increase immune suppression in the TME following infusion CAR T cells present up to 2 mo T-cell expansion and infiltration in some areas of tumor
Goff et al., 2019 [58] (NCT01454596)	18 pts with recurrent GBM		CAR: 3rd-generation CART Route: IV Dosing: 6.3 mil to 23 bil cells LD: yes +Post-infusion IL-2	Median PFS 1.3 mo, one outlier at 12.5 mo Median OS 6.9 mo, 1 pt alive at 59 mo, 2 for 13 mo No OR’s defined by serial MRI	1 mortality at highest dose 2 respiratory symptoms Grade 2 neurological A/E in 10 pts	Median time between biopsy proving EGFRvIII+ and infusion was 11 mo Dose correlated with persistence but not survival CAR T present at 3 mo
Durgin et al., 2021 [174] (NCT02209376)	1 pt		See O’Rourke et al. [57] above	OS 34 mo	Post-infusion D7: flu-like symptoms, including arthralgia, myalgia, and headache	CAR T persistence > 29 mo
Ongoing	Criteria		Intervention	Status	Start	Completion
NCT03296696 Amgen	30 pts with newly diagnosed or recurrent GBM		TCE: AMG 596—EGFRvIII × CD3 BiTE +/− Pembrolizumab Route/dosing: IV continuous	Completed	August 2018	August 2021
NCT04903795 Duke University	18 pts with newly diagnosed GBM or first recurrence		TCE: hEGFRvIII-CD3 Bi-scFv Dosing: 57.0 ng/kg, 570.0 ng/kg, 5700.0 ng/kg, and 57,000.0 ng/kg.	Not yet Recruiting	August 2023	December 2023
NCT05187624 Hoffman-LaRoche	Est 200 pts with newly diagnosed GBM		TCE: RO7428731—EGFRvIIIxCD3 IgG-like Route/dosing: IV Q3 weeks	Recruiting	April 2022	February 2025
NCT03726515 University of Pennsylvania	7 pts with newly diagnosed MGMT-unmethylated GBM		CAR: 2nd-generation, 4-1BB costimulation with Pembrolizumab	Completed—no results	March 2019	February 2021
NCT05063682 (CARTREMENDOUS) Chembrain LTD	10 pts Leptomeningeal disease from EGFRvIII+ GBM		CAR: 2nd-generation EGFRvIII-specific hinge-optimized CD3 ζ-stimulatory/41BB-co-stimulatory Route: IVT x1 +/− additional cycles	Active, not recruiting	May 2020	October 2023
NCT03283631 (INTERCEPT) Duke Uni./NCI	2 pts with recurrent glioblastoma		CAR: EGFRvIII CAR T cells radiolabeled with 111Indium (111In) Route: ITu delivery by CED post-SRS. SPECT on day 1 and 2 to visualize cells Dosing: 250 mil cells	Suspended April 2020 to amend for enrollment of fewer pts Terminated June 2021 to shift to next iteration of a CAR T cell	May 2018	June 2020
NCT02844062 Beijing Sanbo Brain Hospital	Est. 10 pts		CAR: EGFRvIII CAR T cells with truncated EGFR (for tracking/ablation) Route: IV LD: yes	Unknown	July 2016	July 2019 Last updated July 2016
NCT02664363 (ExCeL) Duke University	3 pts		CAR: EGFRvIII CAR radiolabeled with 111Indium Dosing: after resection, SOC and up to 3 cycles of dose-intensified TMZ	Terminated—study funding ended	February 2017	September 2019
NCT05660369 (INCIPIENT) Massachusetts General Hospital	Est. 21 pts with newly diagnosed or recurrent glioblastoma, supratentorial		CAR: CARv3-TEAM-E; T cells transduced with lentiviral vector to express EGFRvIII-CAR and EGFRwt-TEAM Route: IVT (Ommaya reservoir) Dosing: Safety run of one infusion then dose escalation × 3 arms, weekly infusions × 6	Recruiting	March 2023	June 2026
NCT05024175 Massachusetts General Hospital	Est. 18 pts who have completed 24 mo since CARv3-TEAM-E T-cell infusion or <24 mo if they discontinued due to progression or other		Observational study Long-term safety and efficacy of CARv3-TEAM-E T-cell therapy	Not yet recruiting	December 2021	August 2039
NCT05802693 Beijing Tsinghua Chang Gung Hospital	Est 22 pts with recurrent glioblastoma		Ommaya reservoir	Not yet recruiting	April 2023	April 2025
HER2						
Ahmed et al., 2017 [81] (NCT01109095)	17 adult and pediatric pts with rGBM, CMV seropositive		CAR: Autologous 2nd-generation (CD28) HER 2 CAR VST Route: IV Dosing: Dose escalation up to 1 × 10^8^ cells/m^2^, up to 6 doses at 6–12 week intervals at the same dose level LD: no	Median OS 11.1 mo post-CART 3 pts stable at 29, 28.8 and 24 mo. 18 m-survival 29.4% For 7 pts who failed only 1st line Rx: Median OS 27.2 mo 18 m-survival 43%	No DLTs Grade 2 headache/seizure in 2 pts	Median time to infusion 12.5 months 59% had failed 1–5 lines of Rx other than initial SOC No expansion of CAR T but persistence up to 1 year MRI inflammatory responses mimicking progression Survival correlated with lack of previous salvage therapy
Vitanza et al., 2021 [82] (NCT03500991)	3 pts aged 15–26 with recurrent CNS tumors (1 grade 3 astrocytoma, 2 ependymoma)		CAR: 2nd-generation (4-1BB) Route: IC or IVT Dosing: 3 doses per month for a max of 18 doses, dose 10 mil to 25 mil LD: no	PD in 2/3 pts	No DLTs H/A, pain at spinal met site, worsening neurologic deficit IV route: fever	High CRP with symptoms
Ongoing	Criteria/enrollment		CAR and administration	Status	Start	Completion
NCT03389230 City of Hope	Est. 42 pts with recurrent/refractory grade 3 or 4 glioma		CAR: 2nd gen. HER2-Specific, Hinge-Optimized, 41BB-Costimulatory and Truncated CD19 Route/dosing: IC or ITu or both weekly for 3 weeks	Recruiting	August 2018	December 2023
EphA2						
Lin et al., 2021 [87] (NCT03423992)	3 pts (initial cohort)		CAR: 2nd gen EphA2-CAR, 4-1BB costimulatory domain and truncated EGFR Route: IV Dosing: single infusion, starting at 1 mil cells/kg LD: yes (Flu/cyclo)	SD in 1 pt, PD in 2 pts OS 81-186d	Grade 2 CRS and pulmonary edema in 2/3 patients	Expansion of CAR T cells peripherally persisting for 28d+, with peak at 7–10 d
NKG2D						
Ongoing	Criteria/enrollment		CAR and administration	Status	Start	Completion
NCT05131763 Fudan University	Est 3 pts with relapsed HCC, GBM, medulloblastoma, or colon Ca		CAR: 2nd gen CAR with 4-1BB costim Route: IV	Recruiting	March 2021	December 2023
NCT04717999 UWELL Biopharma	20 pts with 1st or 2nd GBM relapse		NKG2D CAR-T Route: Ommaya reservoir	Not yet recruiting	September 2021	December 2023
B7-H3						
Tang et al., 2021 [92]	1 patient with rGBM and 50% heterogeneous B7-H3 expression		CAR: B7-H3 CAR-T Route: IC Dosing: 4 mil to 20 mil cells Weekly	PR after first cycle Symptomatic and MRI PD after cycle 6	No DLT Grade 2 headache related to infusions worse in the first 4 cycles	Expansion of T cells and CART in the CSF, peak cycle 3, and decline in later cycles
Ongoing	Criteria/enrollment		CAR and administration	Status	Start	Completion
NCT04385173 Zhejiang University	12 pts with recurrent or refractory GBM		Route: ITu, IVT Dosing: 3 injections, 1–2 week intervals In between temozolomide cycles	Recruiting	December 2022	May 2024
NCT04077866 Zhejiang University	40 pts with recurrent or refractory GBM		2 arms, B7H3 CART + temozolomide vs. temozolomide alone Route: ITu, IVT Dosing: 3 injections, 1–2 week intervals In between temozolomide cycles	Recruiting	June 2023	August 2025
GD2						
Liu et al., 2023 [175] NCT03170141	8 adult and pediatric pts with recurrent IDH-WT, MGMT unmethylated, GBM		CAR: 4th gen CAR-T cells (CD28 transmembrane and cytoplasmic domains, co-stimulatory 4-1BB, CD3z, IC9) Route: Single IV (*n* = 5) or IV + ICT if surgical candidates (*n* = 3) Dose: 2.5 mil cells/kg (IV), 100,000 cells/Kg (ICT) LD: yes (cyclo + fludara)	PR in 4/8, SD in 1/8 and PD in 3/8 on 28-day MRI Median OS 10 mo (3–24 mo)	No DLT 1 grade 3 headache 1 grade 2 seizure	CART expansion, peak at 1–3 weeks. CART detected in all pts at 4 weeks. Pts with PD were alive at 6–24 mo post-infusion; 1 confirmed treatment effect rather than TP at Bx. Antigen loss Increased CD8+ T cells, decreased M2 macrophages
Ongoing	Criteria/enrollment		CAR and administration	Status	Start	Completion
NCT04099797 (GAIL-B) Baylor College of Medicine	34 pts with High grade glioma, DIPG, embryonal or ependymal tumors		CAR: C7R-GD2.CART Route: Ommaya reservoir/VP shunt Dose: 10 mil to 30 mil cells/m^2^ LD: yes (cyclo/fludara)	Recruiting	February 2020	Primary: February 2025 Final: February 2039
Ongoing	Target and Criteria/enrollment		CAR and administration	Status	Start	Completion
NCT05577091 Beijing Tiantan Hospital	CD133 CD44 [176]	Est. 10 patients with recurrent glioblastoma	CAR: 4^th^-gen dual-target truncated IL7Ra modified CAR T cells Route: Ommaya reservoir ITu Dosing: low-dose group and high-dose group receiving 1 dose, multidose-group receiving weekly infusions × 8 weeks max	Not yet Recruiting	May 2023	Primary: November 2024 Final: November 2032
NCT03423992 Xuanwu Hospital, Beijing	EGFRVIII, IL13Rα2, Her-2, EphA2, CD133, GD2	Est 100 pts	CAR: Autologous T cells expressing CAR ± PD-L1 antibody	Recruiting	March 2018	January 2023 Last updated June 2021
NCT04214392 City of Hope	Chlorotoxin	36 pts with MMP2+ recurrent for progressive GBM	CAR: Chlorotoxin (EQ)-CD28-CD3zeta-CD19t-expressing CAR T Route/dosing: Arm 1: single delivery, 3 weekly cycles: begins with 1 infusion intracranial intratumoral or intracavitary (ICT) and lasts for 1 week. Arm 2: dual delivery ICT and intraventricular	Recruiting	February 2020	December 2024
NCT04045847 Xijing Hospital	CD147	31 pts with Recurrent GBM	CAR: CD147-CART Route: Ommaya reservoir Dosing: 3 doses at weekly intervals	Unknown	May 2019	May 2022 Last updated May 2022
NCT05353530 (IMPACT) University of Florida	CD70	Newly diagnosed MGMT unmethylated GBM	CAR: Autologous IL-8 receptor (CXCR2) modified CD70 CAR (8R-70CAR) Route: IV Dose: Single infusion 2 weeks after RT of 1 mil–100 mil cells LD: in 1 cohort	Not yet recruiting	October 2022	December 2025 (primary completion) December 2040
NCT03170141 Shenzhen Geno-Immune Medical Institute	Multiple (GD2, EGFRvIII, CD70)	Est 20 pts with recurrent GBM	CAR: EGFRvIII targeting CAR-T cells modified with immune modulatory genes (IgT) i.e., ICI Route: IV or ITu in 3 days (split dose) Dose: 50,000/kg to 25 mil/kg LD: yes (fludara and/or cyclo)	Enrolling by invitation	May 2020	December 2023

Abbreviations: cyclo: cyclophosphamide; DLT: dose-limiting toxicity; fludara: fludarabine; IC: intracavitary; IV: intravenous; IVT: intraventricular; LD: lymphodepletion; LM: Leptomeningeal; PD: progressive disease; PR: partial response; SD: stable disease; TEAM: T-cell-engaging antibody molecule; TP: true progression. * Information obtained through clinicaltrials.gov accessed in March 2023.
